# Supplementary material for: Sesquiterpene Coumarins, Chromones, and Acetophenone Derivatives with Selective Cytotoxicities from the Roots of Ferula caspica M. Bieb. (Apiaceae)
Source: Pharmaceuticals (Basel). 2024 Sep 24;17(10):1254. doi: 10.3390/ph17101254 (PMC11509956; doi:10.3390/ph17101254)
Supplement: Supplementary file 1 [file pharmaceuticals-17-01254-s001.zip › pharmaceuticals-3193354-supplementary.pdf]

Supplementary Materials

Sesquiterpene Coumarins, Chromones, and Acetophenone Derivatives with Selective Cytotoxicities from the Roots of *Ferula caspica* M. Bieb. (Apiaceae)

Fadıl Kaan Kuran[1,4], Gülsüm Altıparmak Ülbegi[2], Gülşah Gamze Arcan[2], Fatma Memnune Eruçar[1], Şule Nur Karavuş[3], Pınar Aksoy Sağırlı[2], Nur Tan[1], and Mahmut Miski[1*]

[1]Istanbul University, Faculty of Pharmacy, Department of Pharmacognosy, 34116 Istanbul, Türkiye

[2] Istanbul University, Faculty of Pharmacy, Department of Biochemistry, 34116 Istanbul, Türkiye

[3] Istanbul Medipol University, School of Pharmacy, Department of Pharmacognosy, 34810 Istanbul, Türkiye

[4]Istanbul University, Institute of Graduate Studies in Health Sciences , Department of Pharmacognosy, 34116 Istanbul, Türkiye

* Correspondence: Prof. Dr. Mahmut Miski; e-mail: miski@comcast.net

Table of Contents

Figure S1. Bioactivity-guided isolation scheme for the cytotoxic sesquiterpene compounds of the dichloromethane root extract of *Ferula caspica* 4
Figure S2. ^{1}H NMR spectrum (500 MHz, $CDCl_3$) of kayserin A (**1**) 5
Figure S3. ^{13}C APT NMR spectrum (125 MHz, $CDCl_3$) of kayserin A (**1**) 6
Figure S4. ^{1}H-^{1}H COSY spectrum ($CDCl_3$) of kayserin A (**1**) 7
Figure S5. HSQC spectrum ($CDCl_3$) of kayserin A (**1**) 8
Figure S6. HMBC spectrum ($CDCl_3$) of kayserin A (**1**) 9
Figure S7. NOESY spectrum ($CDCl_3$) of kayserin A (**1**) 10
Figure S8. (+)-HRESIMS spectrum of kayserin A (**1**) 11
Figure S9. ^{1}H NMR spectrum (500 MHz, $CDCl_3$) of kayserin B (**2**) 12
Figure S10. ^{13}C NMR (APT) spectrum (125 MHz, $CDCl_3$) of kayserin B (**2**) 13
Figure S11. ^{1}H-^{1}H COSY spectrum ($CDCl_3$) of kayserin B (**2**) 14
Figure S12. HSQC spectrum ($CDCl_3$) of kayserin B (**2**) 15
Figure S13. HMBC spectrum ($CDCl_3$) of kayserin B (**2**) 16
Figure S14. NOESY spectrum ($CDCl_3$) of kayserin B (**2**) 17
Figure S15. (+)-HRESIMS spectrum of kayserin B (**2**) 18
Figure S16. ^{1}H NMR spectrum (500 MHz, $CDCl_3$) of 8′-*epi*-kayserin B angelate (**3**) ... 19
Figure S17. ^{13}C NMR spectrum (125 MHz, $CDCl_3$) of 8′-*epi*-kayserin B angelate (**3**) .. 20
Figure S18. ^{1}H-^{1}H COSY spectrum ($CDCl_3$) of 8′-*epi*-kayserin B angelate (**3**) 21
Figure S19. HSQC spectrum ($CDCl_3$) of 8′-*epi*-kayserin B angelate (**3**) 22
Figure S20. HMBC spectrum ($CDCl_3$) of 8′-*epi*-kayserin B angelate (**3**) 23
Figure S21. NOESY spectrum ($CDCl_3$) of 8′-*epi*-kayserin B angelate (**3**) 24
Figure S22. (+)-HRESIMS spectrum of 8′-*epi*-kayserin B angelate (**3**) 25
Figure S23. ^{1}H NMR spectrum (500 MHz, $CDCl_3$) of 3-*epi*-ferulin D (**4**) 26
Figure S24. ^{13}C NMR spectrum (125 MHz, $CDCl_3$) of 3-*epi*-ferulin D (**4**) 27
Figure S25. ^{1}H-^{1}H COSY spectrum ($CDCl_3$) of 3-*epi*-ferulin D (**4**) 28
Figure S26. HSQC spectrum ($CDCl_3$) of 3-*epi*-ferulin D (**4**) 29
Figure S27. HMBC spectrum ($CDCl_3$) of 3-*epi*-ferulin D (**4**) 30
Figure S28. NOESY spectrum ($CDCl_3$) of 3-*epi*-ferulin D (**4**) 31
Figure S29. (+)-HRESIMS spectrum of 3-*epi*-ferulin D (**4**) 32
Figure S30. ^{1}H NMR spectrum (500 MHz, $CDCl_3$) of ferulin D (**5**) 33
Figure S31. ^{1}H NMR spectrum (500 MHz, $CDCl_3$) of 7-desmethylferulin D (**6**) 34
Figure S32. ^{1}H NMR spectrum (500 MHz, $CDCl_3$) of colladonin (**7**) 35
Figure S33. ^{13}C NMR spectrum (125 MHz, $CDCl_3$) of colladonin (**7**) 36
Figure S34. ^{1}H NMR spectrum (500 MHz, $CDCl_3$) of isosamarcandin (**8**) 37

Figure S35. ^{13}C NMR spectrum (125 MHz, $CDCl_3$) of isosamarcandin (**8**) 38
Figure S36. ^{1}H NMR spectrum (500 MHz, $CDCl_3$) of farnesyl furanocoumarin derivative (**9**) 39
Figure S37. ^{1}H NMR spectrum (500 MHz, $CDCl_3$) of 1-(3′-nerolidyl)-4,6-dihydroxyacetophenone (NDHAP) (**10**) 40
Figure S38. ^{1}H NMR spectrum (500 MHz, $CDCl_3$) of 1-hydroxy-1-(1′-farnesyl)-4,6-dihydroxyacetophenone (HFDHAP) (**11**) 41

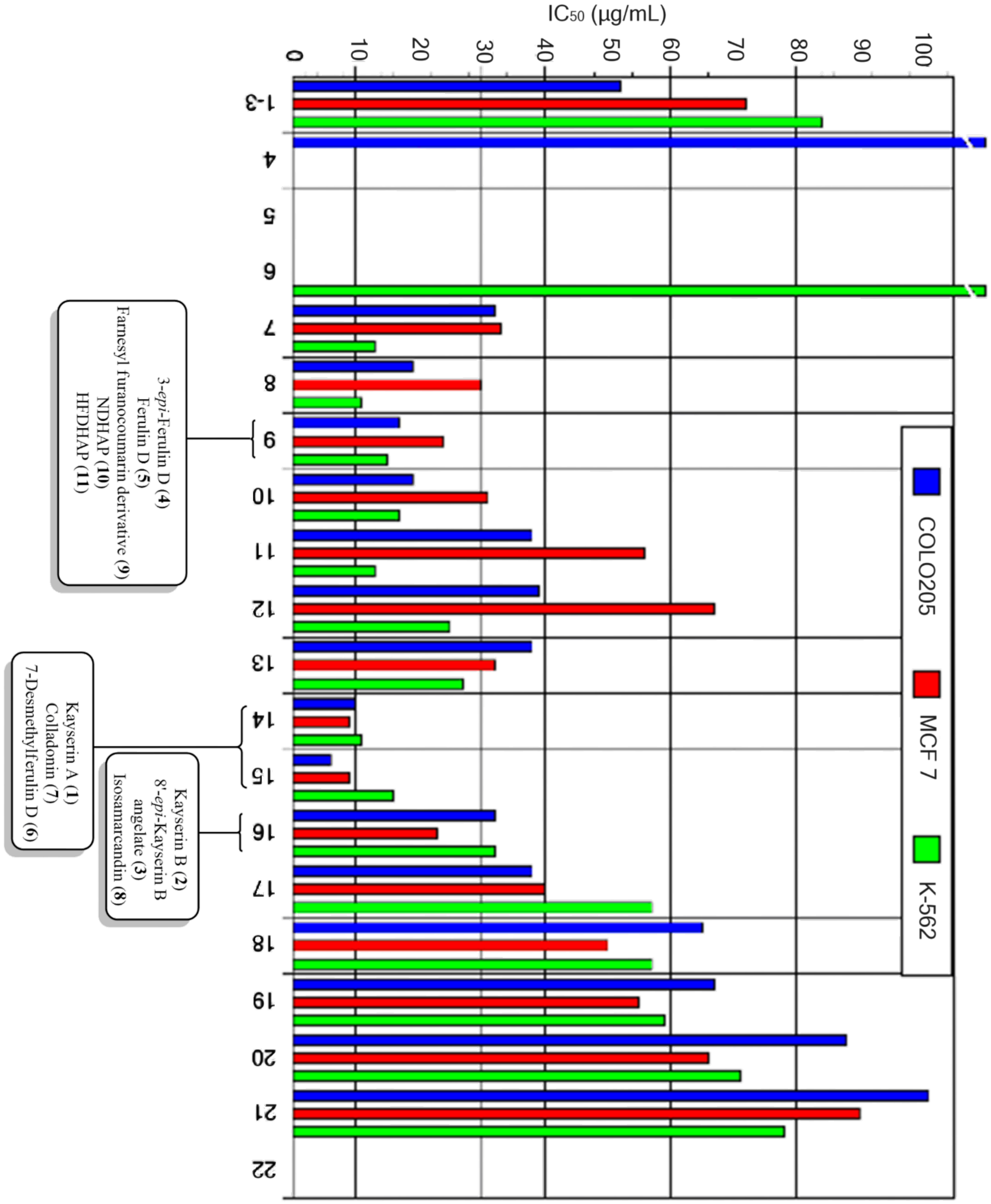

Figure S1. Bioactivity-guided isolation scheme for the cytotoxic sesquiterpene compounds of the dichloromethane root extract of *Ferula caspica*

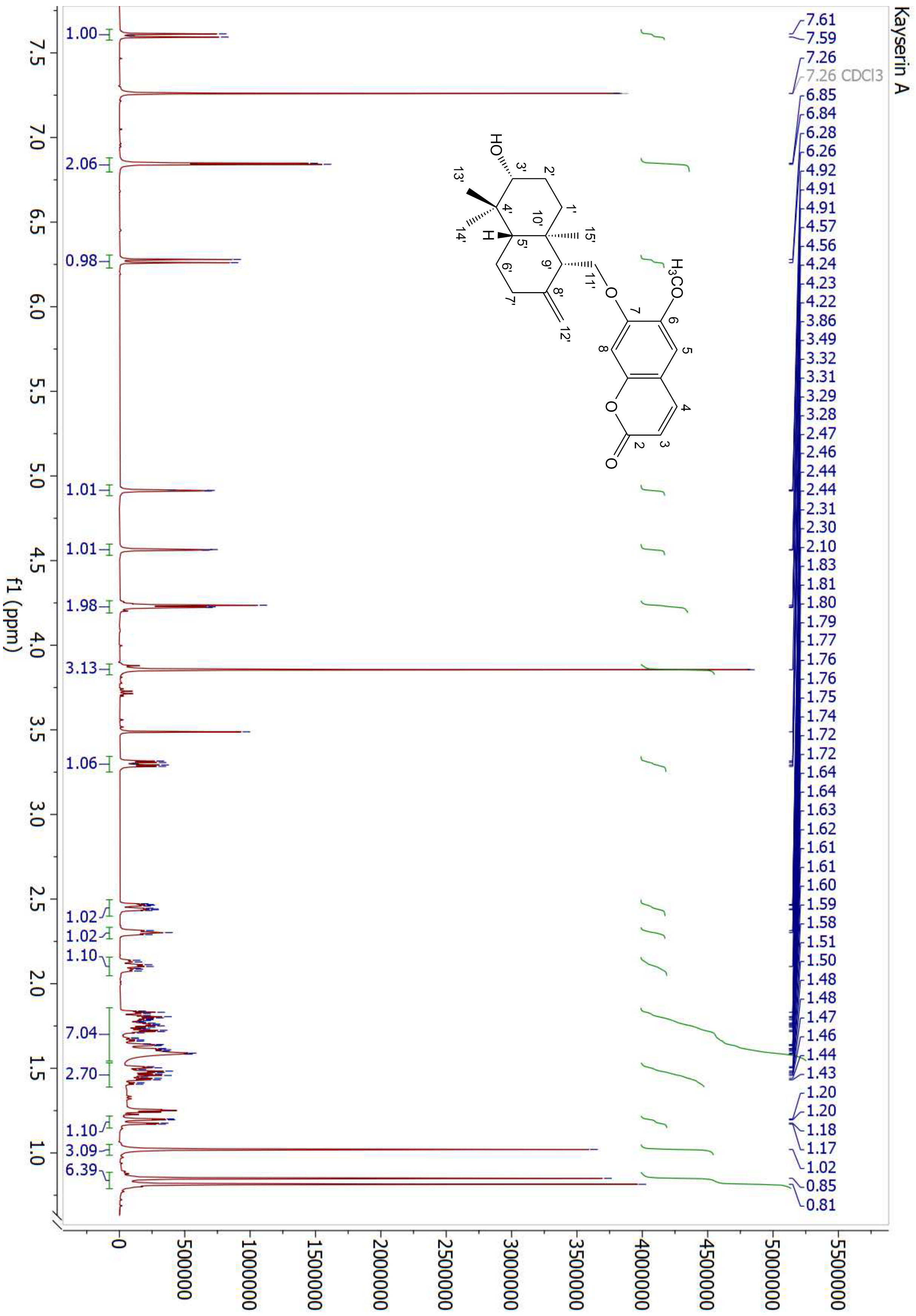

Figure S2. ^{1}H NMR spectrum (500 MHz, $CDCl_3$) of kayserin A (**1**)

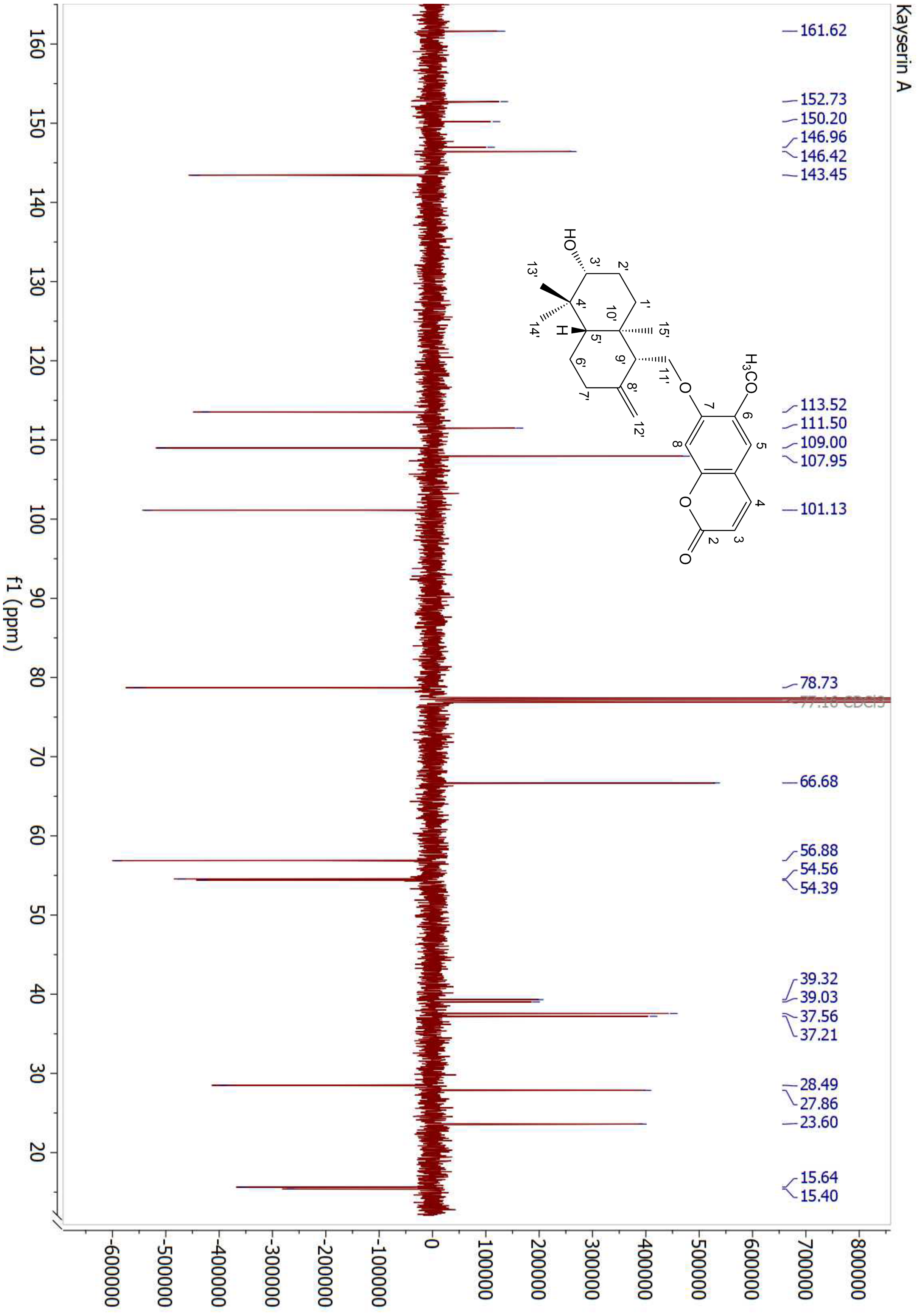

Figure S3. ^{13}C APT NMR spectrum (125 MHz, $CDCl_3$) of kayserin A (**1**)

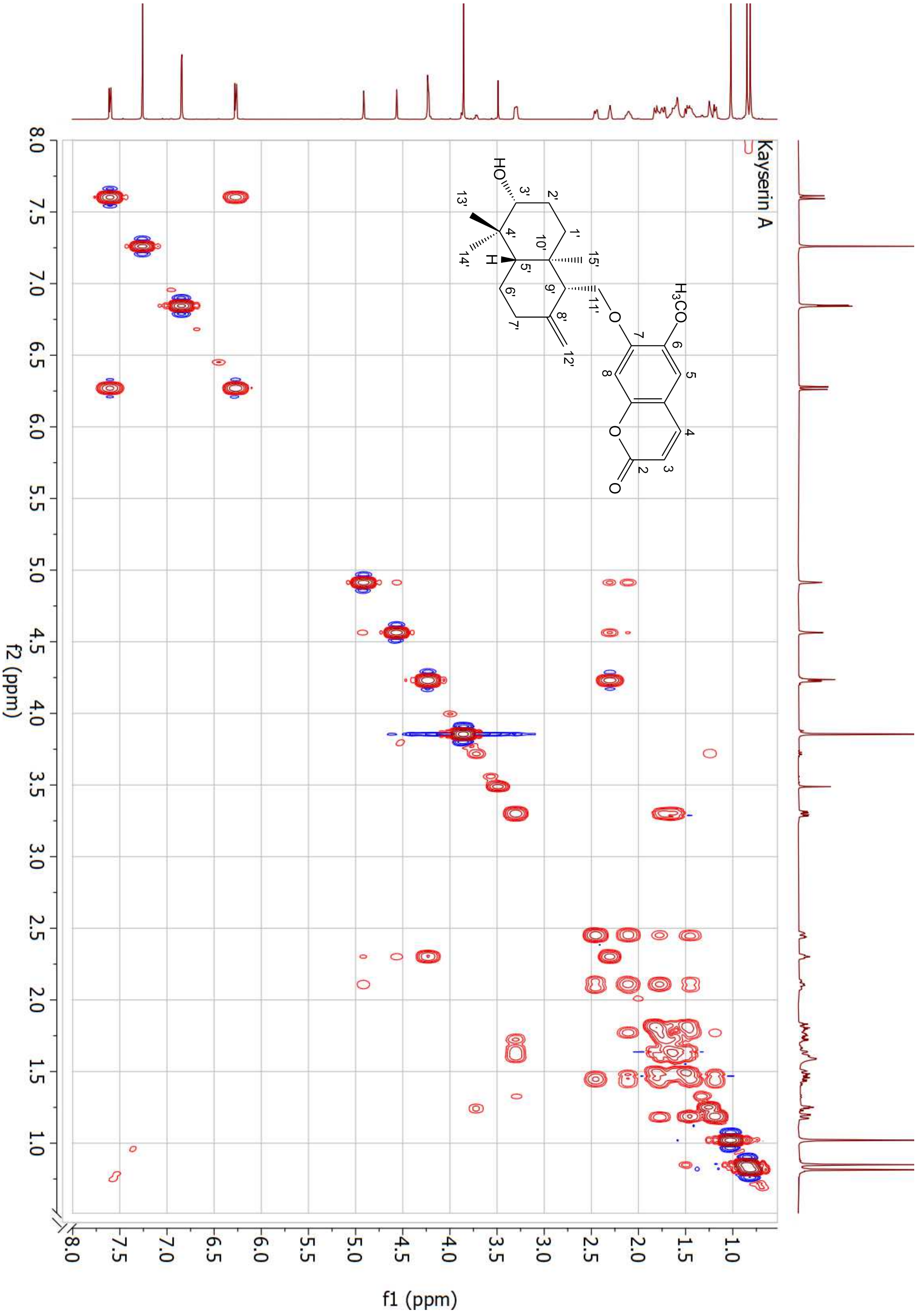

Figure S4. ^{1}H-^{1}H COSY spectrum ($CDCl_3$) of kayserin A (**1**)

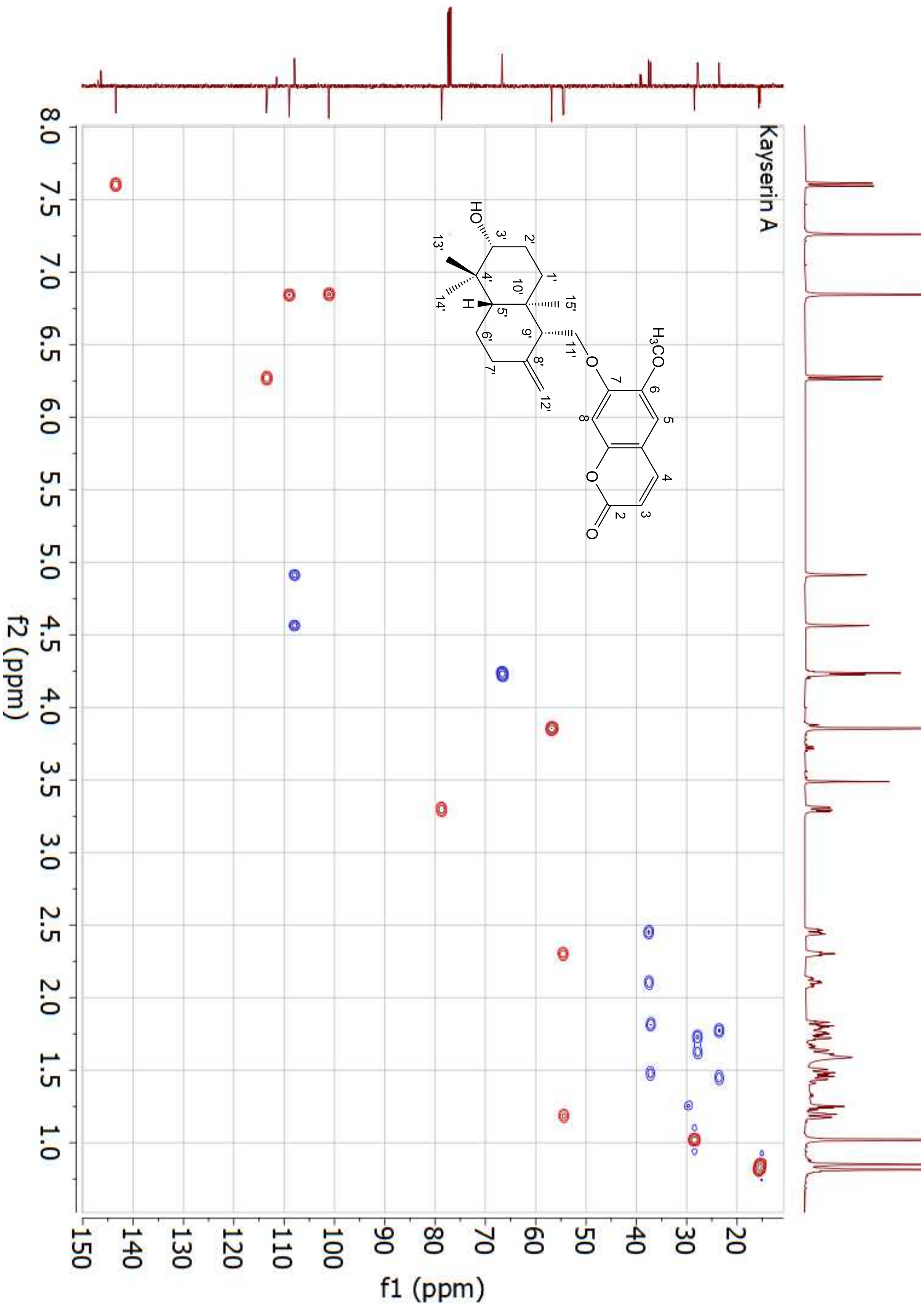

Figure S5. HSQC spectrum ($CDCl_3$) of kayserin A (**1**)

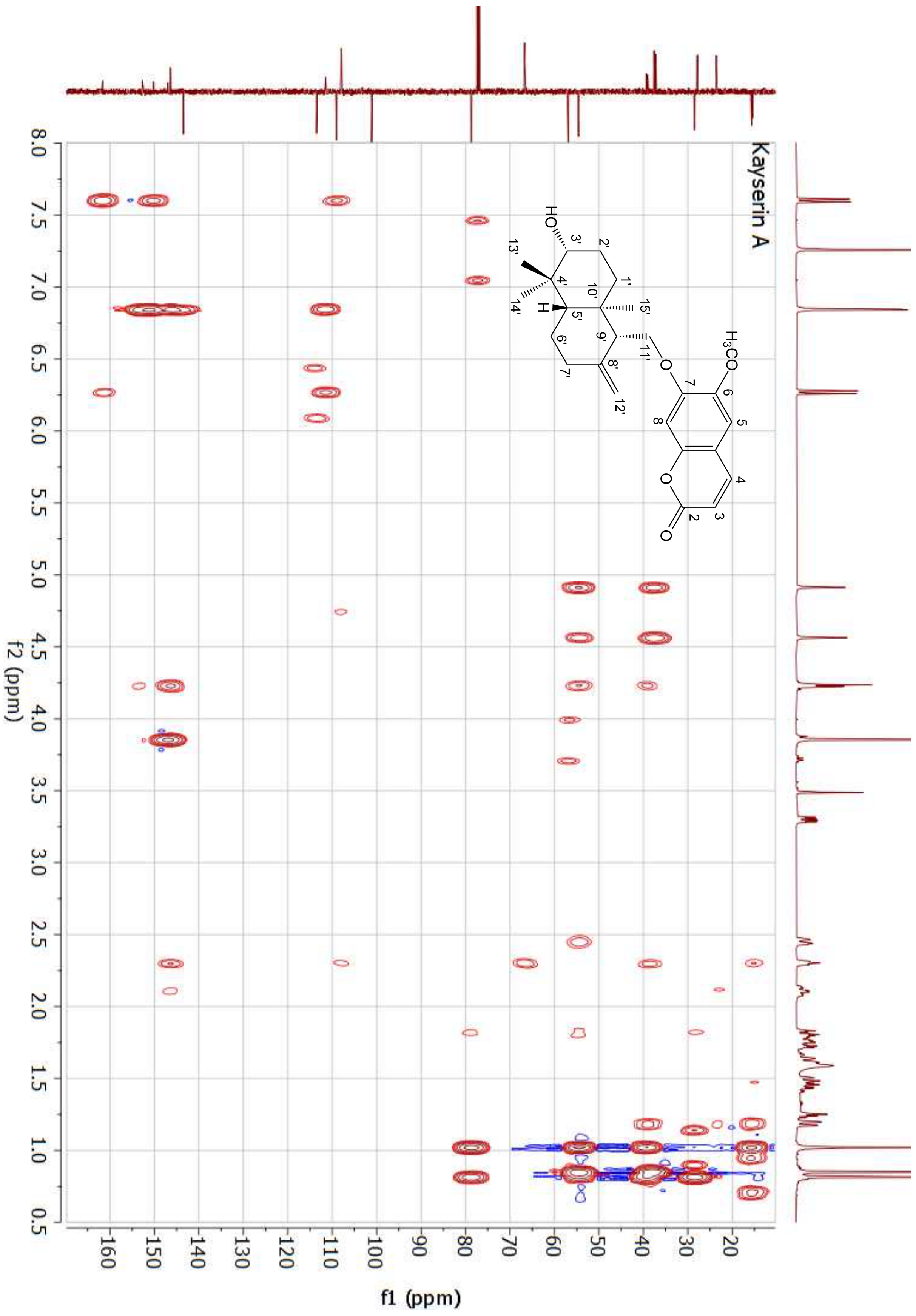

Figure S6. HMBC spectrum ($CDCl_3$) of kayserin A (**1**)

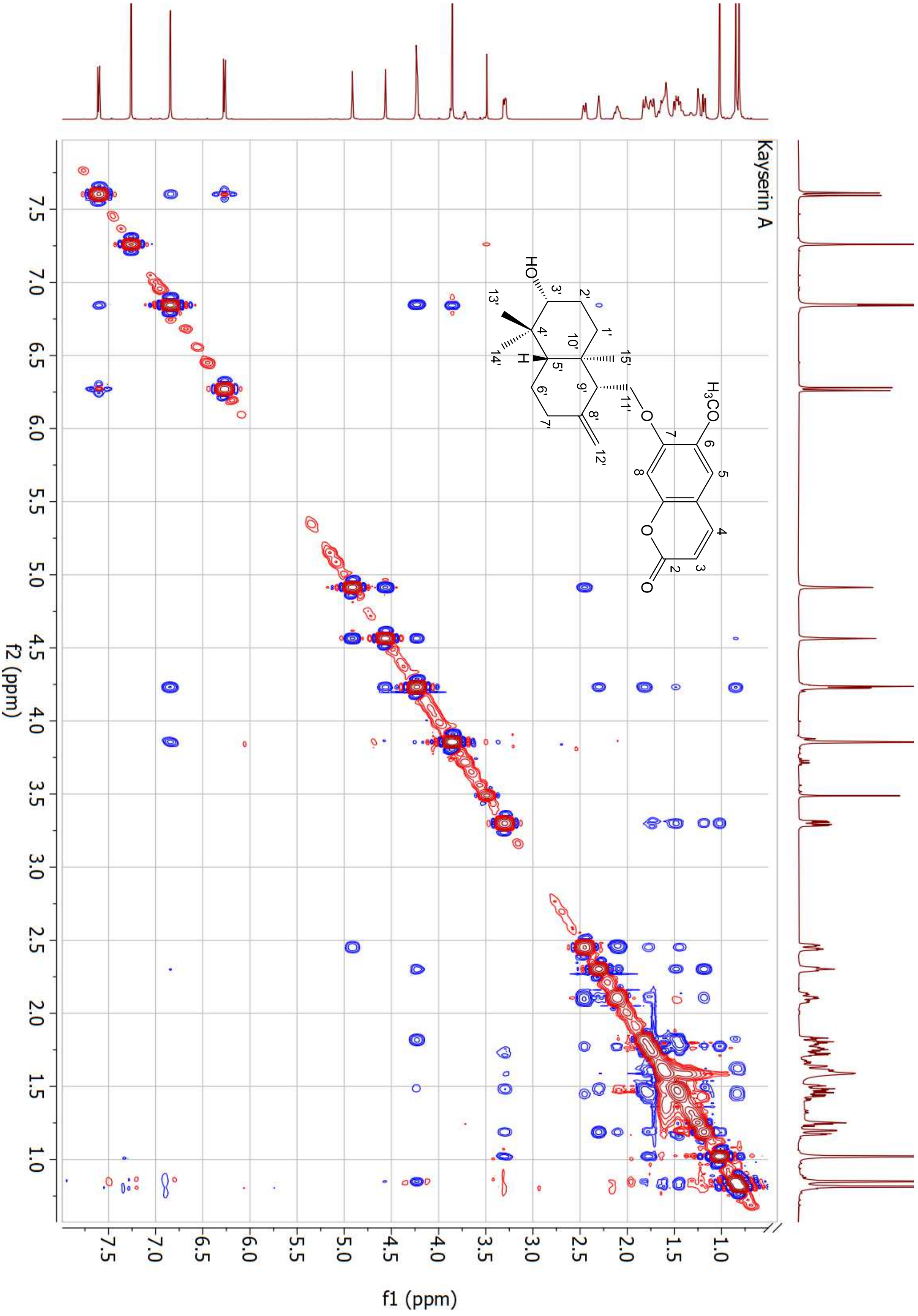

Figure S7. NOESY spectrum (CDCl3) of kayserin A (**1**)

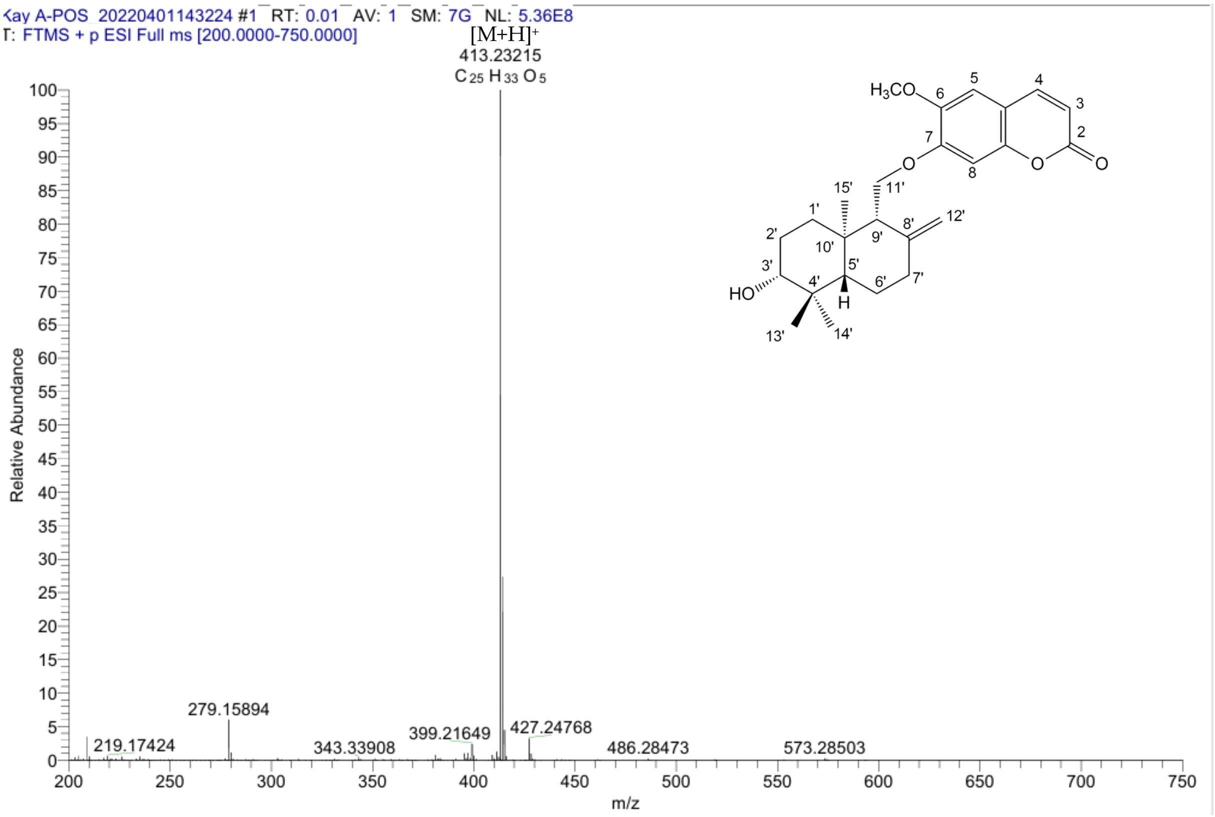

Figure S8. (+)-HRESIMS spectrum of kayserin A (**1**)

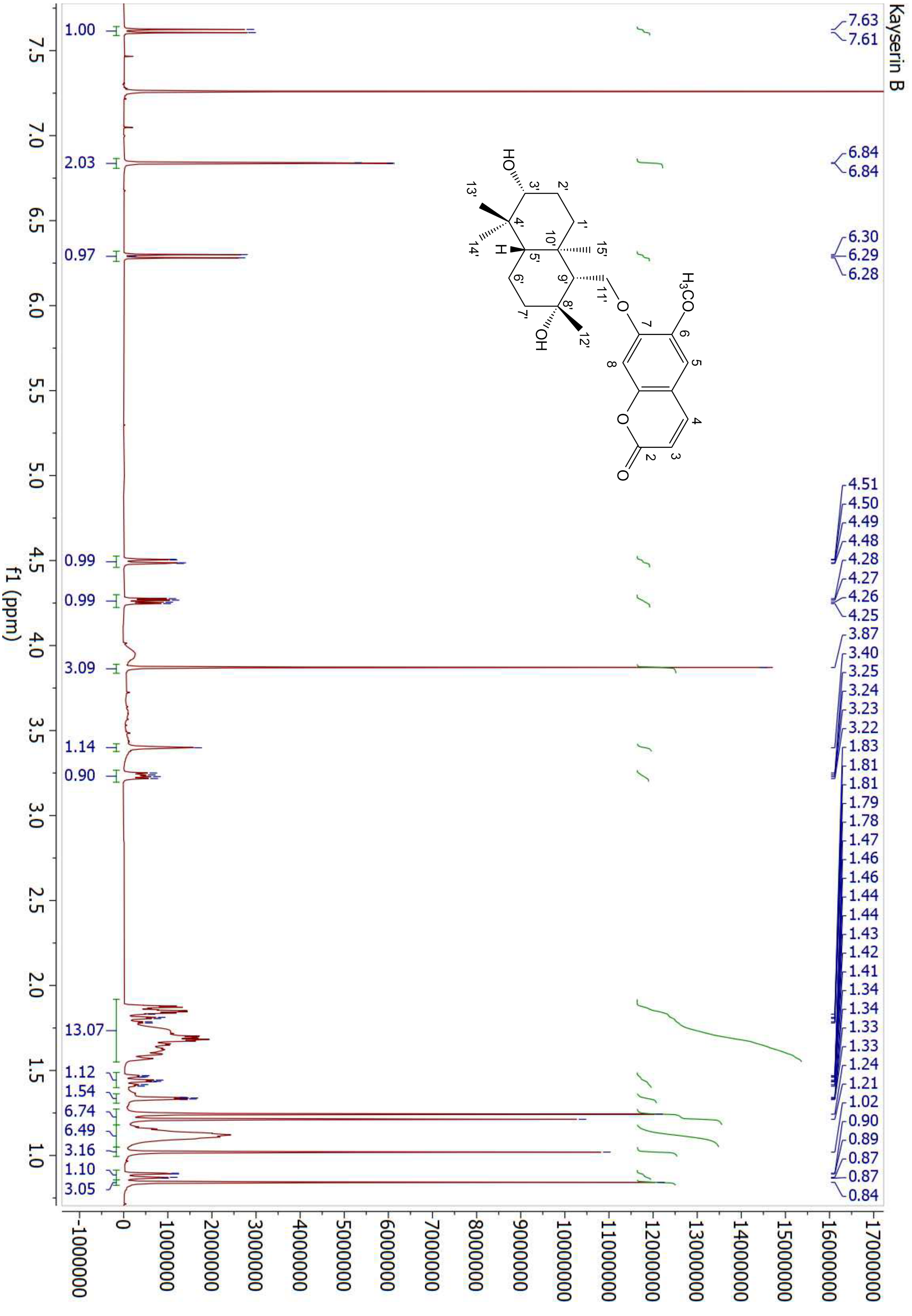

Figure S9. ^{1}H NMR spectrum (500 MHz, $CDCl_3$) of kayserin B (**2**)

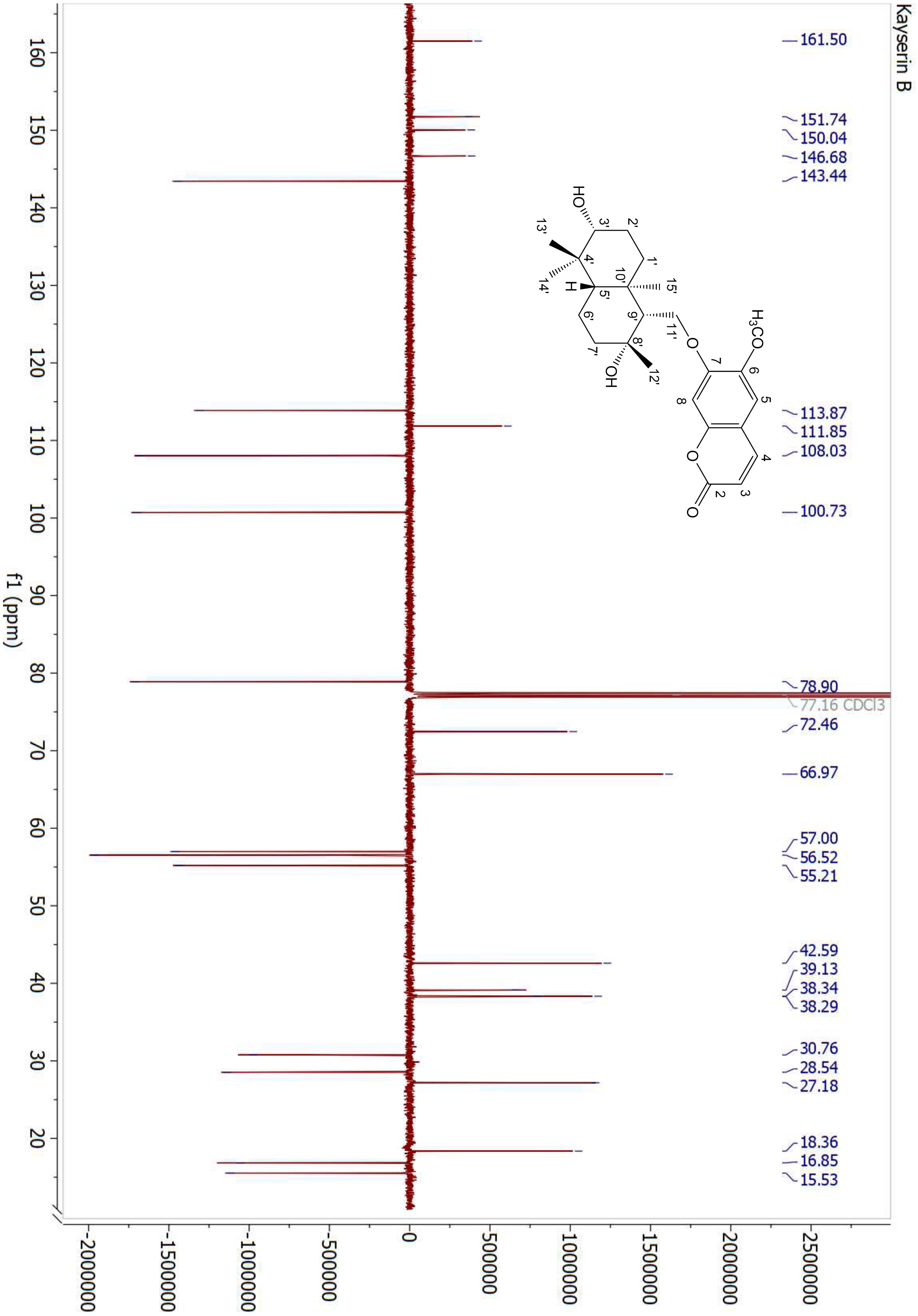

Figure S10. ^{13}C NMR (APT) spectrum (125 MHz, $CDCl_3$) of kayserin B (**2**)

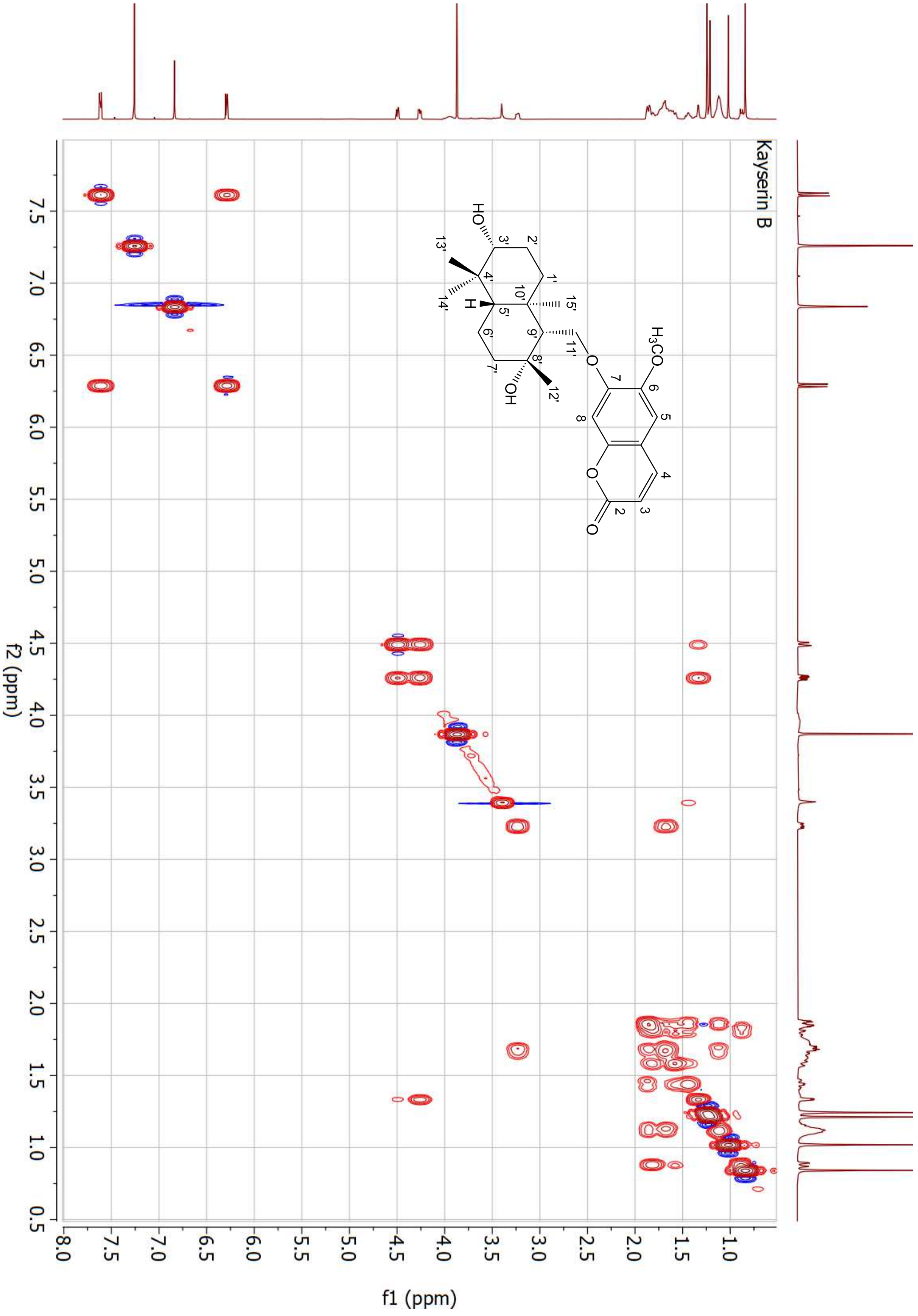

Figure S11. ^{1}H-^{1}H COSY spectrum ($CDCl_3$) of kayserin B (**2**)

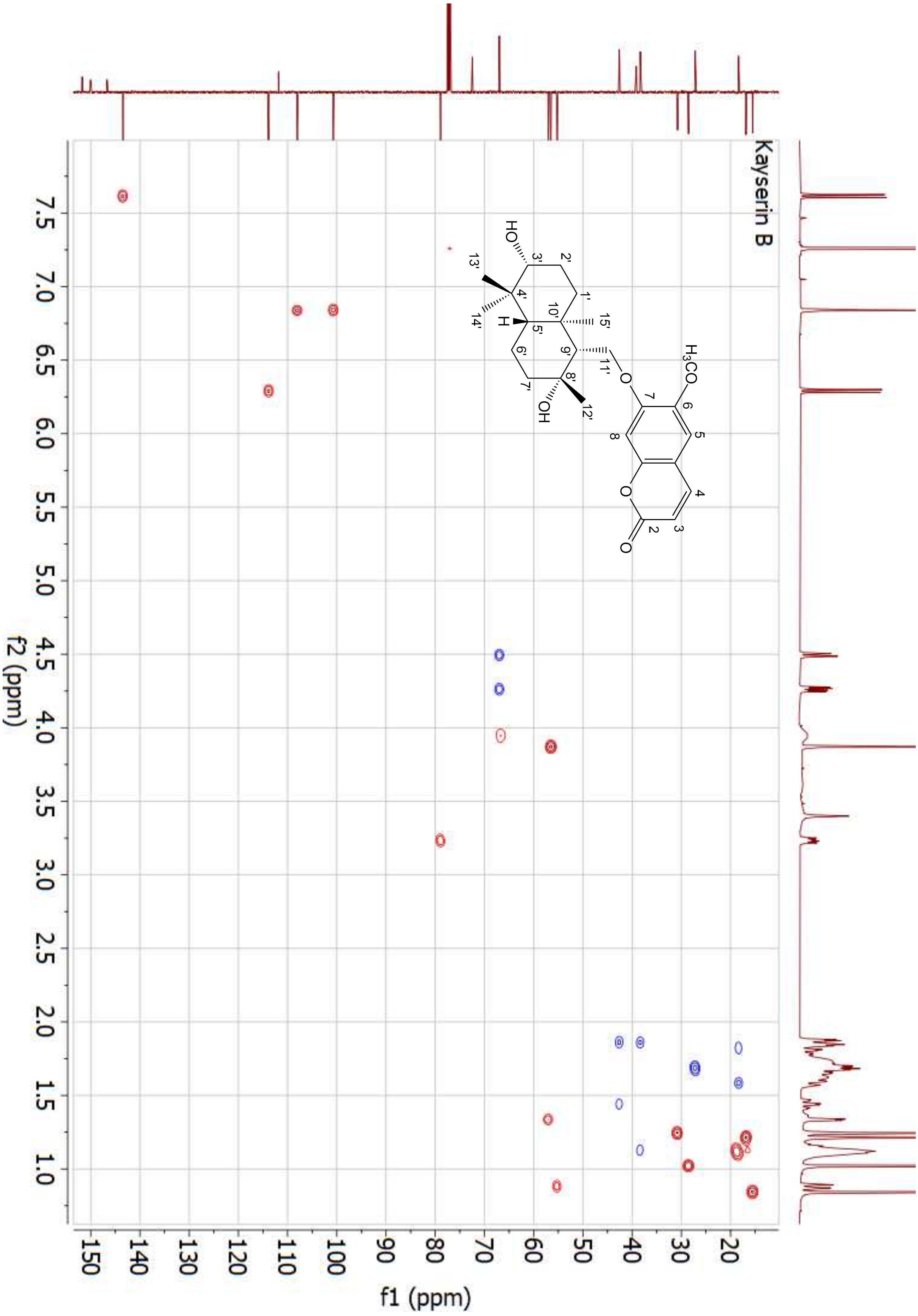

Figure S12. HSQC spectrum ($CDCl_3$) of kayserin B (**2**)

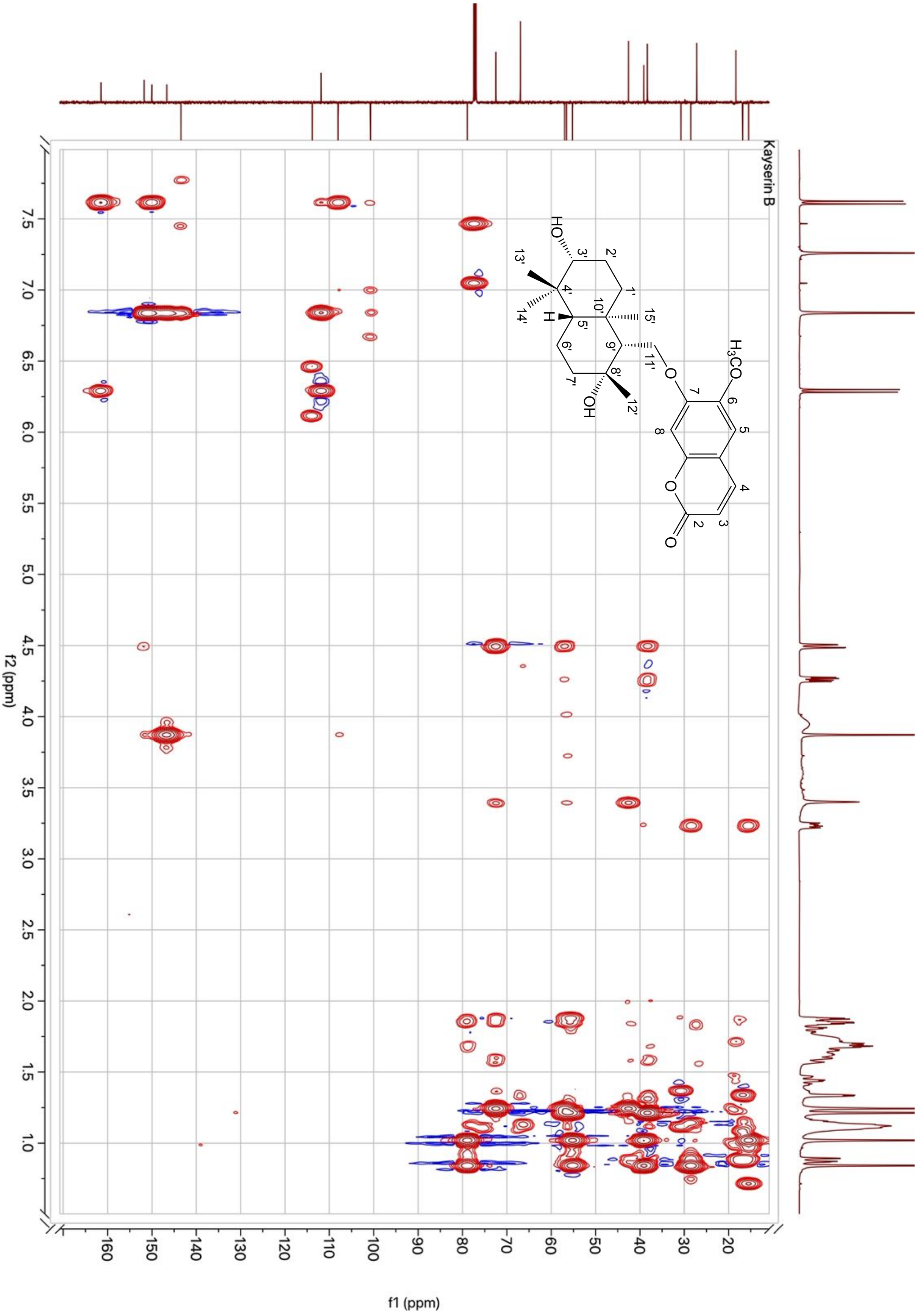

Figure S13. HMBC spectrum ($CDCl_3$) of kayserin B (**2**)

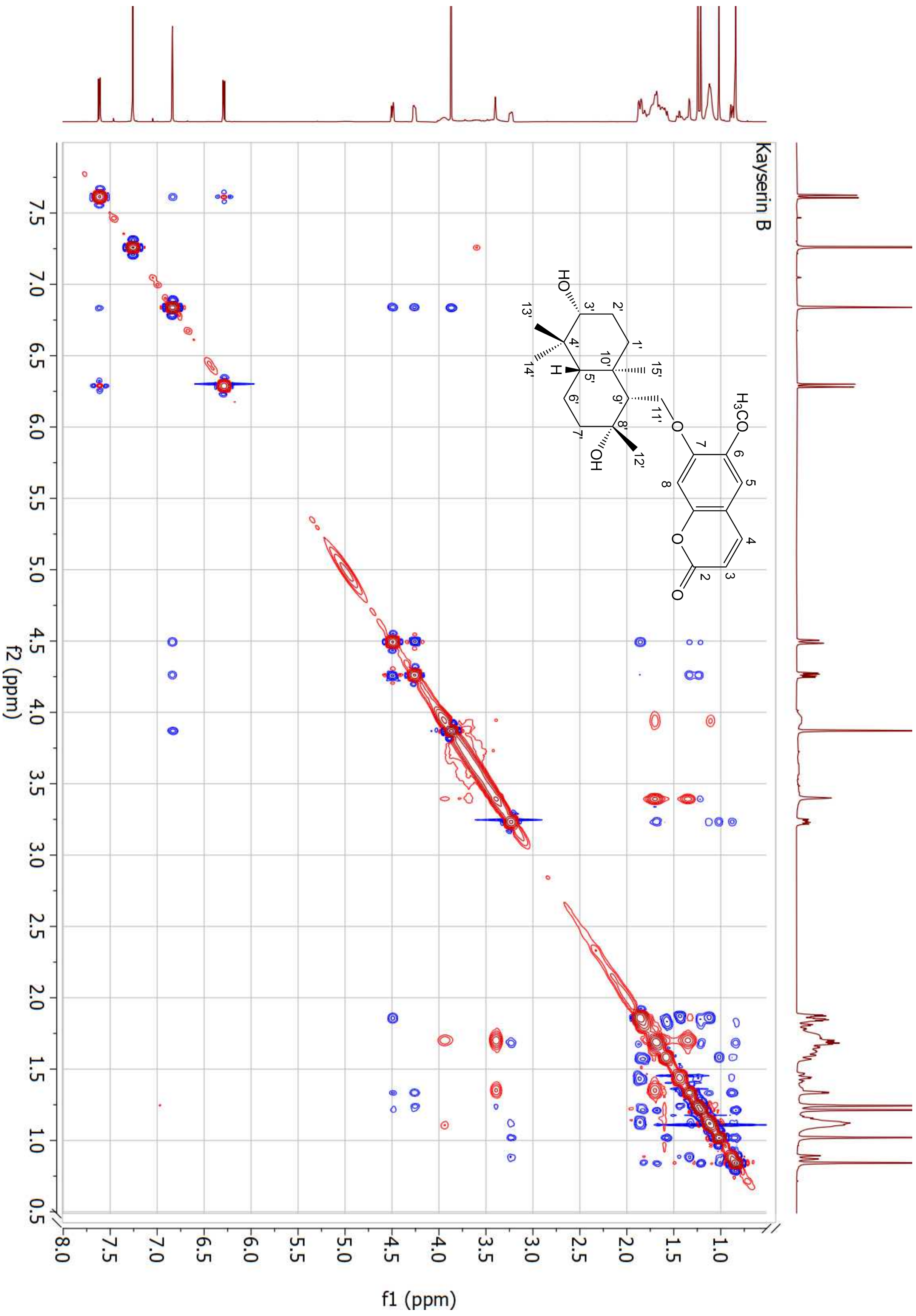

Figure S14. NOESY spectrum ($CDCl_3$) of kayserin B (**2**)

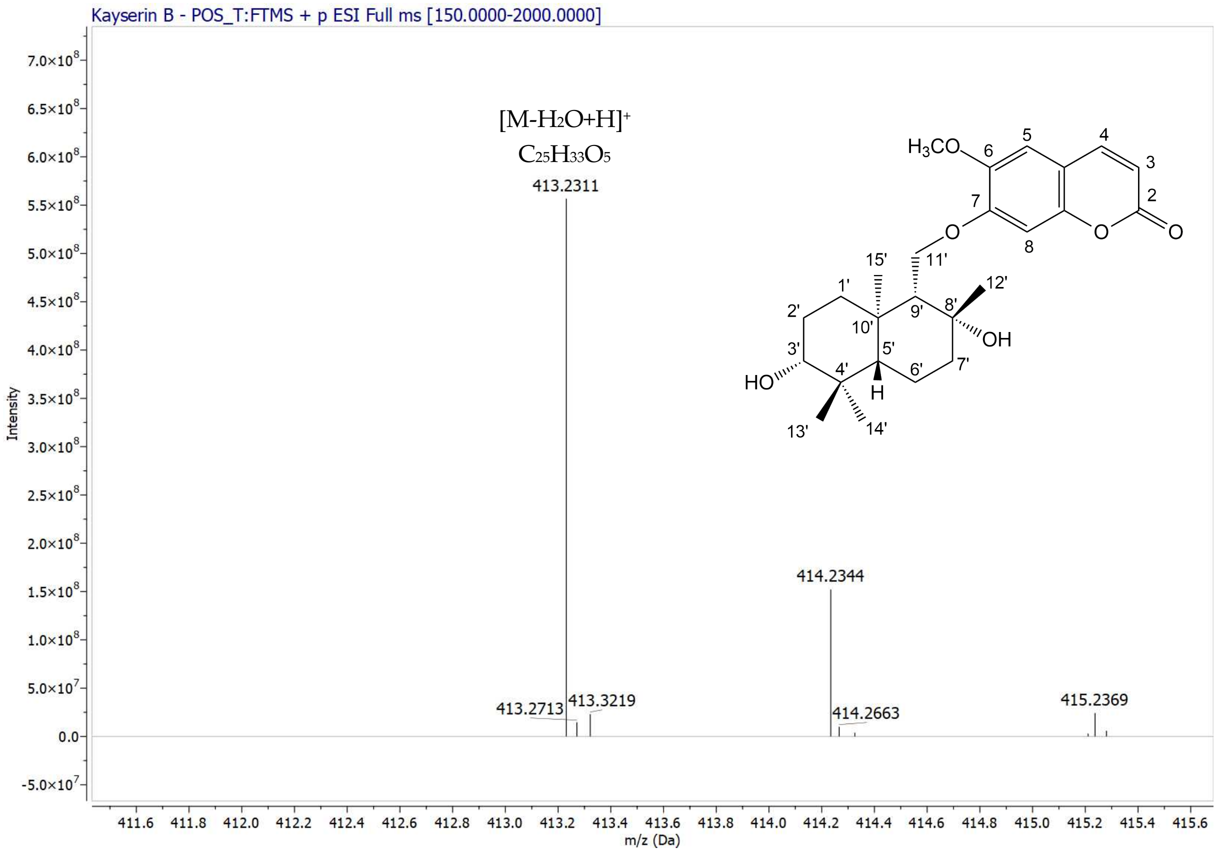

Figure S15. (+)-HRESIMS spectrum of kayserin B (**2**)

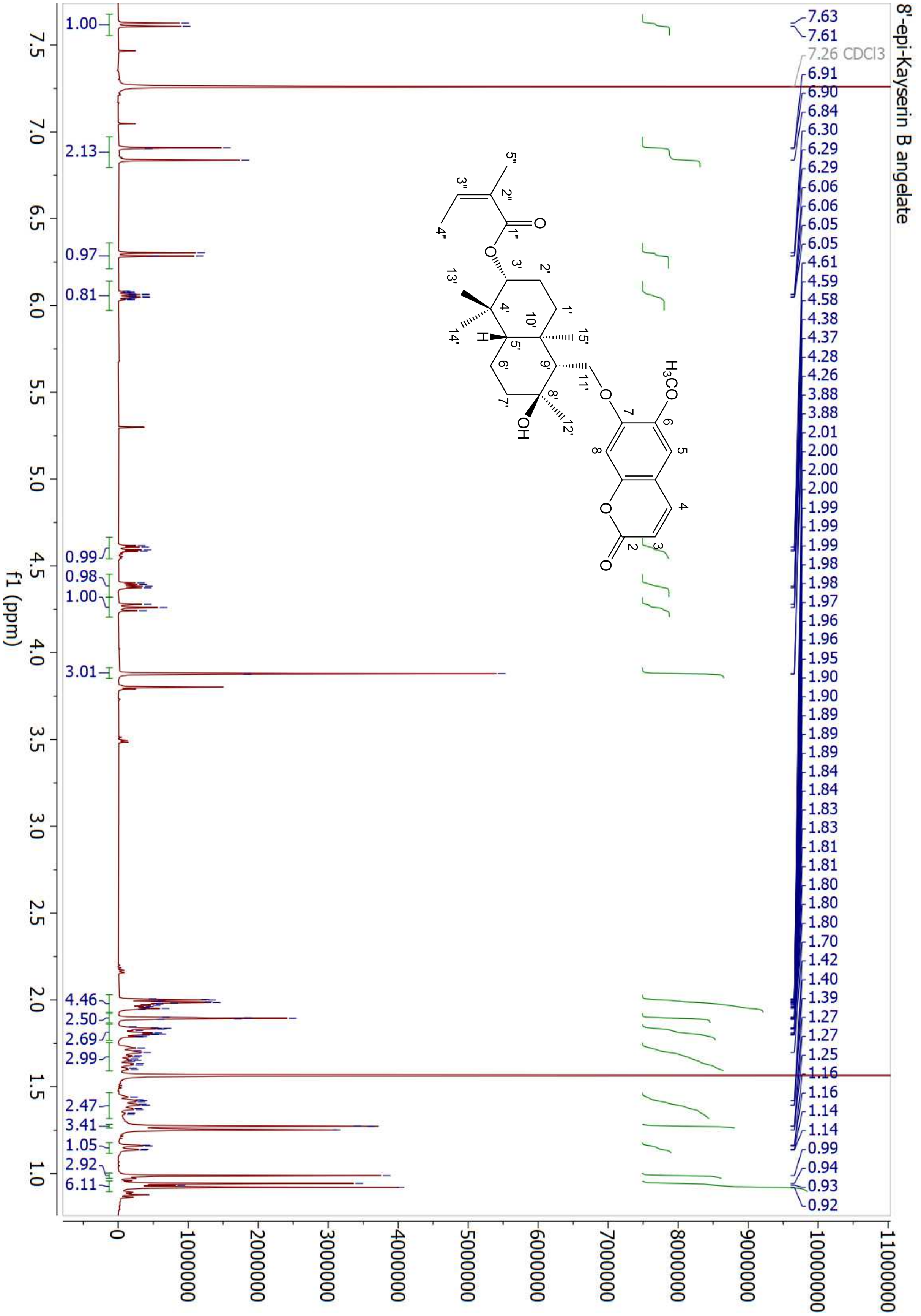

Figure S16. ^{1}H NMR spectrum (500 MHz, $CDCl_3$) of 8′-*epi*-kayserin B angelate (**3**)

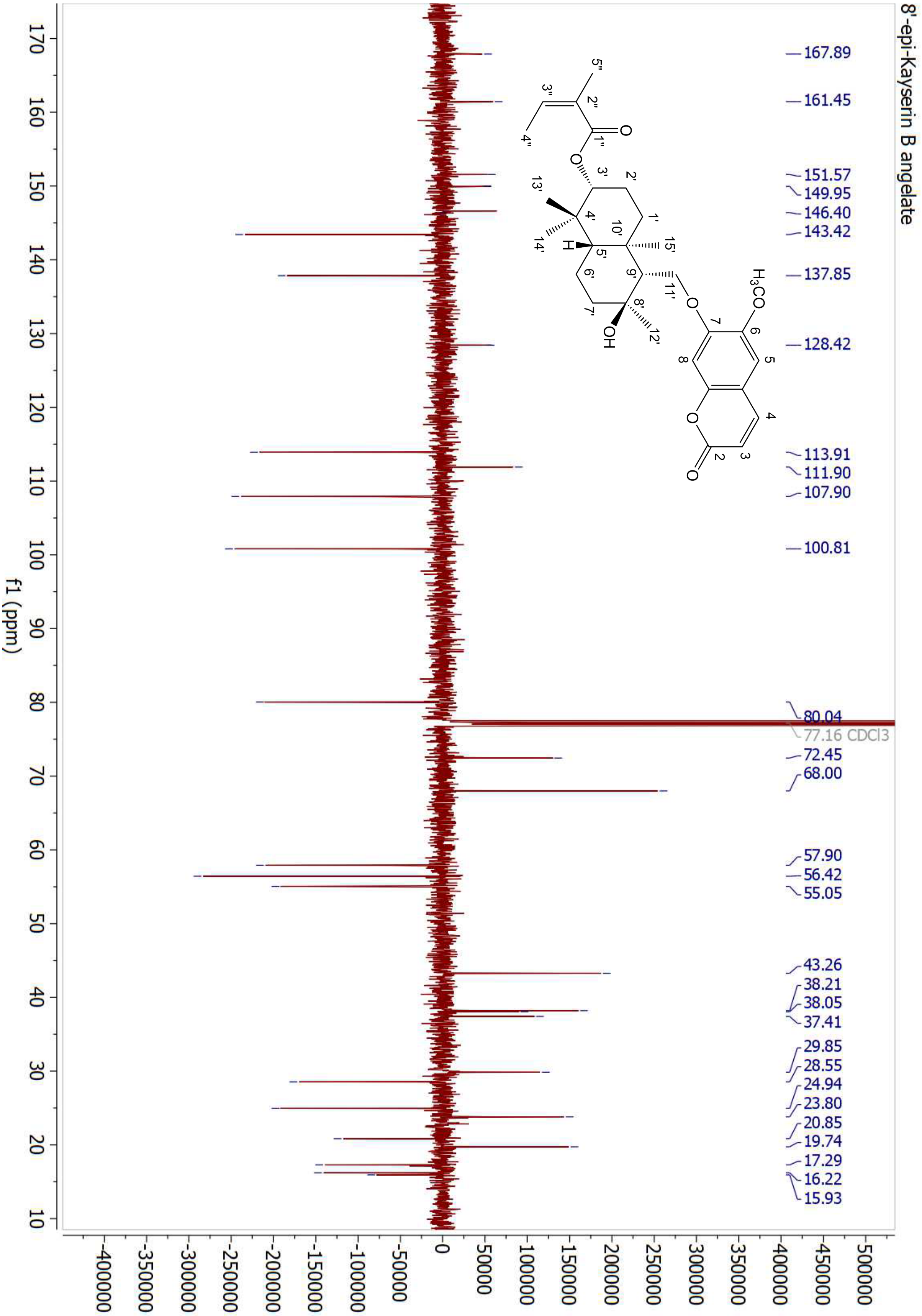

Figure S17. ${}^{13}C$ NMR spectrum (125 MHz, $CDCl_3$) of 8′-*epi*-kayserin B angelate (**3**)

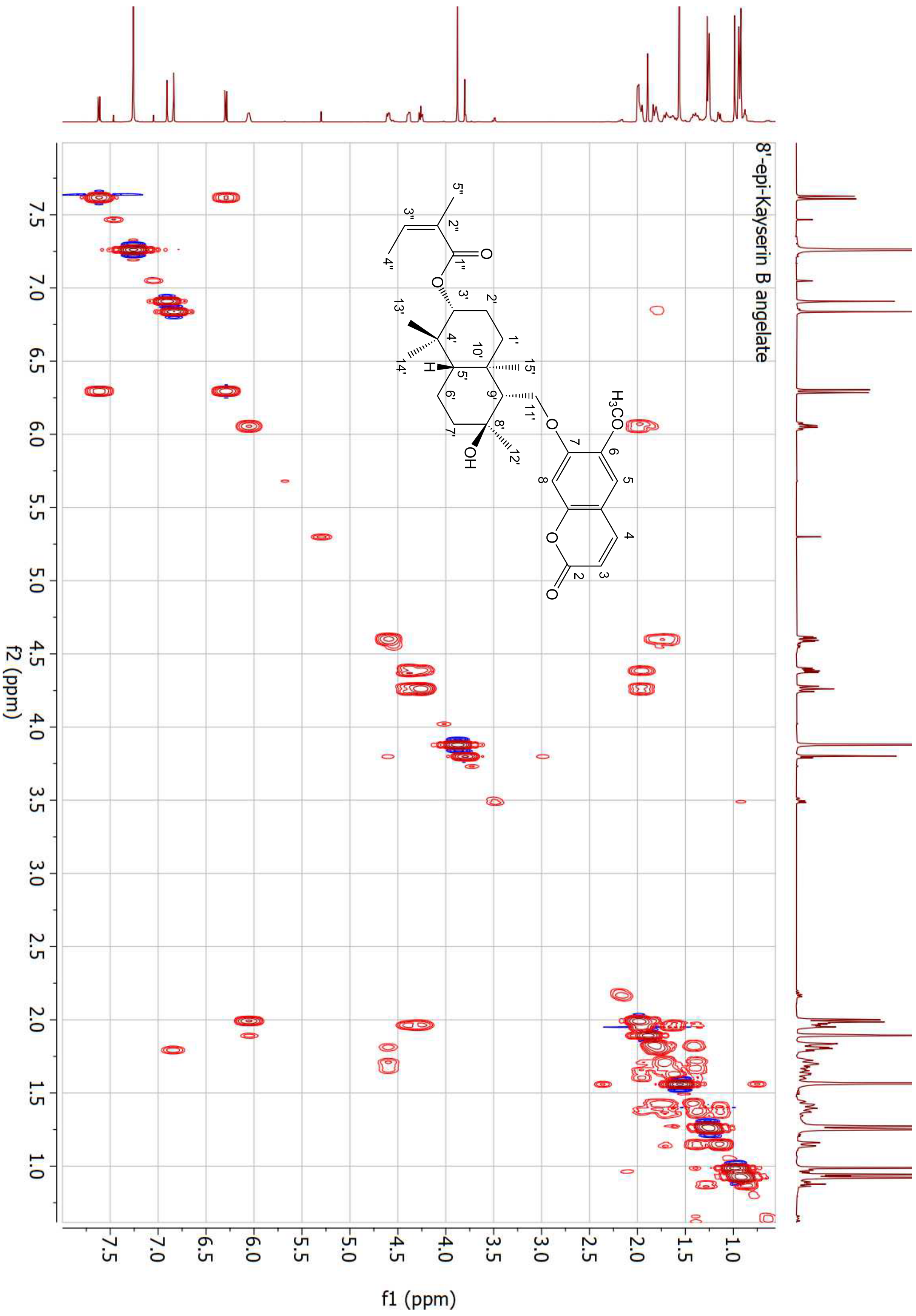

Figure S18. ^1H-^1H COSY spectrum ($CDCl_3$) of 8′-*epi*-kayserin B angelate (**3**)

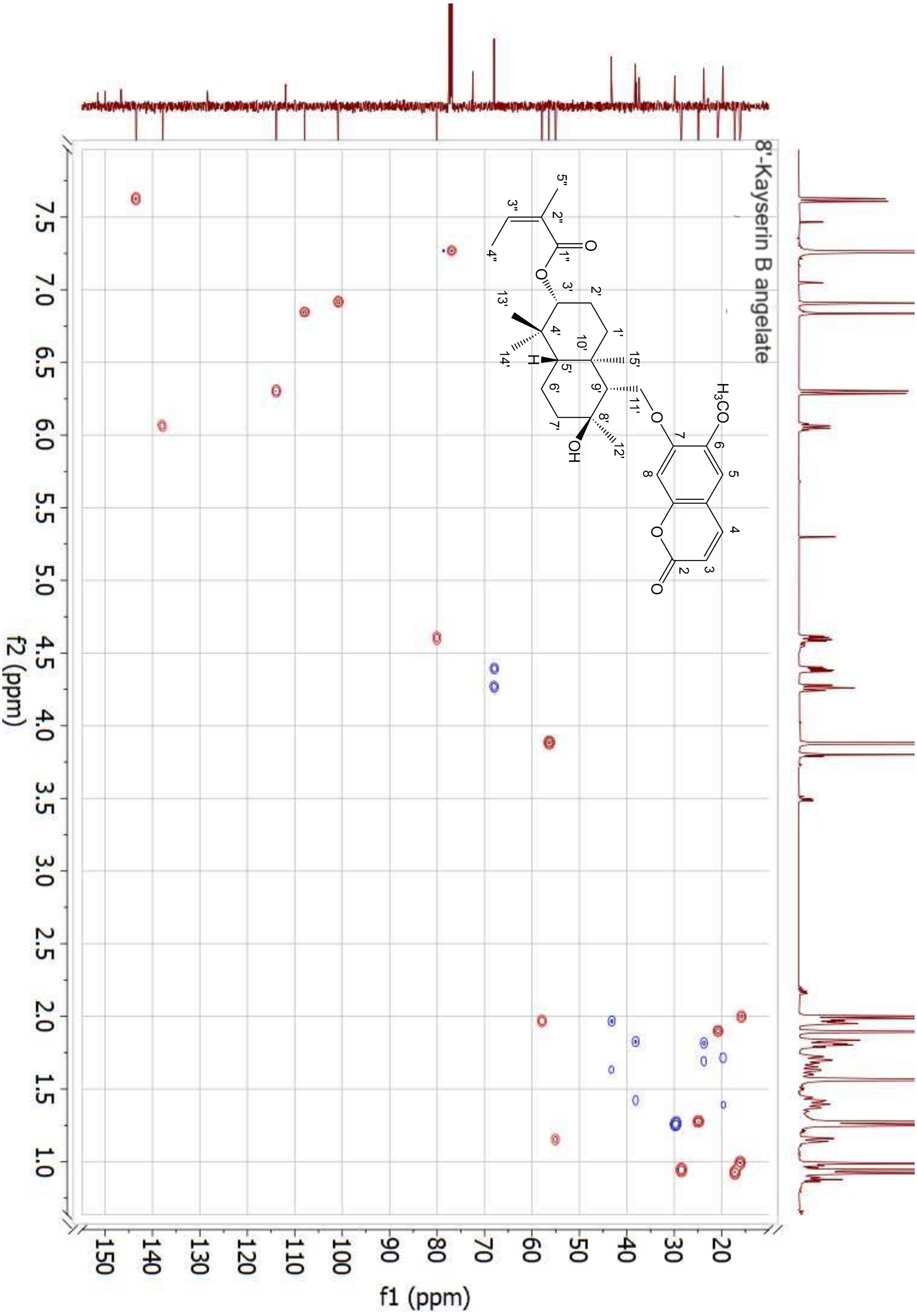

Figure S19. HSQC spectrum ($CDCl_3$) of 8′-*epi*-kayserin B angelate (**3**)

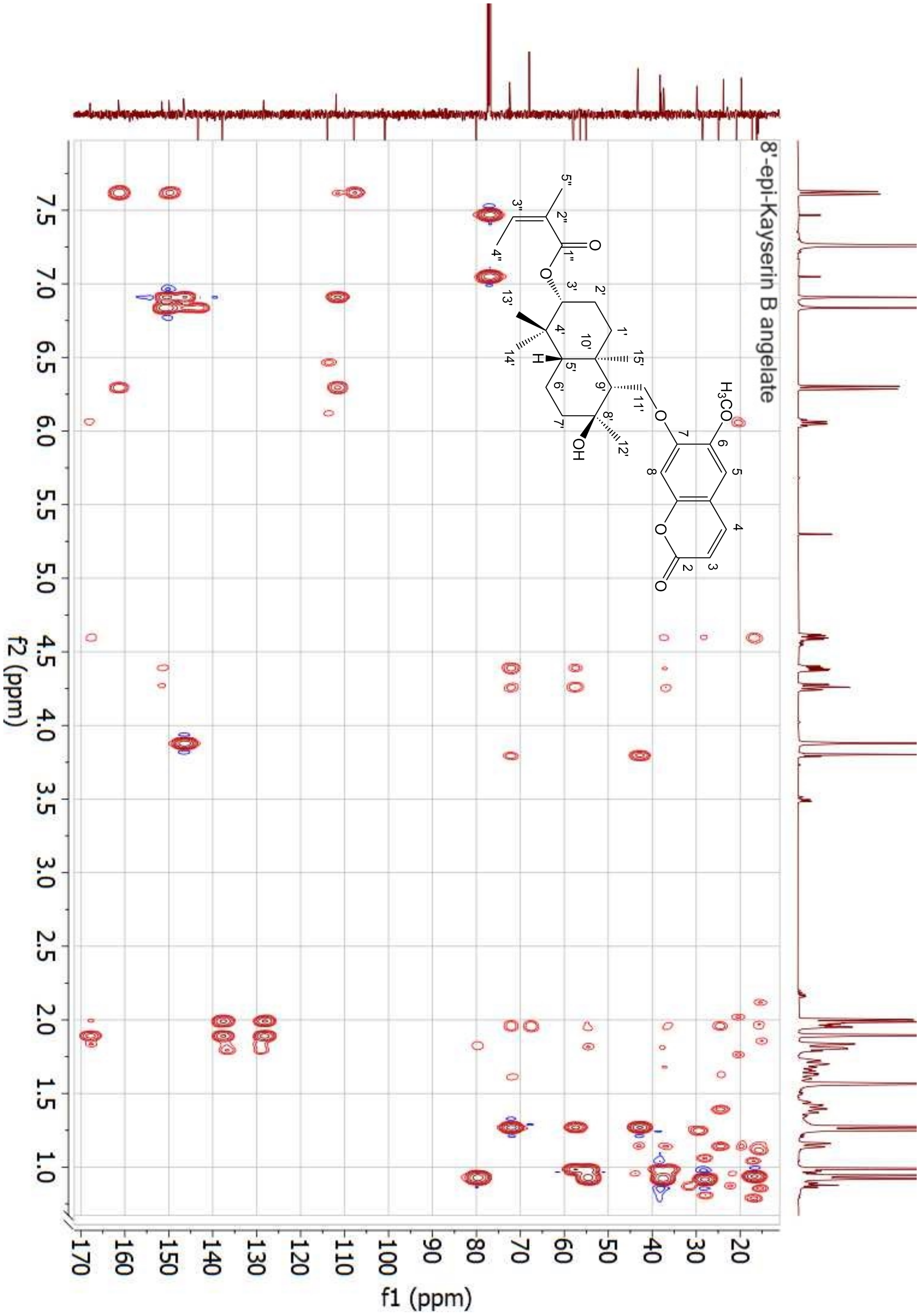

Figure S20. HMBC spectrum ($CDCl_3$) of 8′-*epi*-kayserin B angelate (**3**)

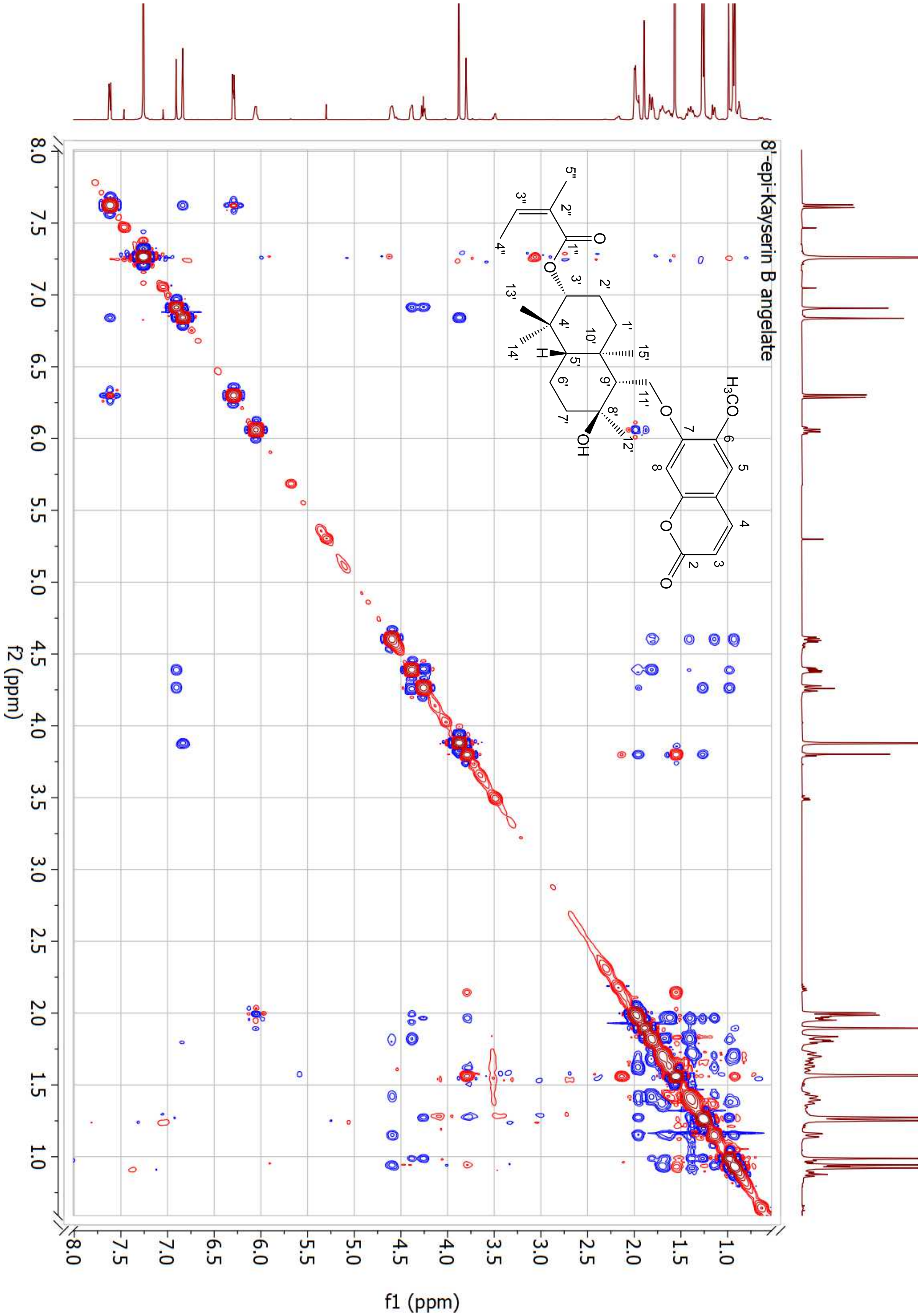

Figure S21. NOESY spectrum (CDCl3) of 8′-*epi*-kayserin B angelate (**3**)

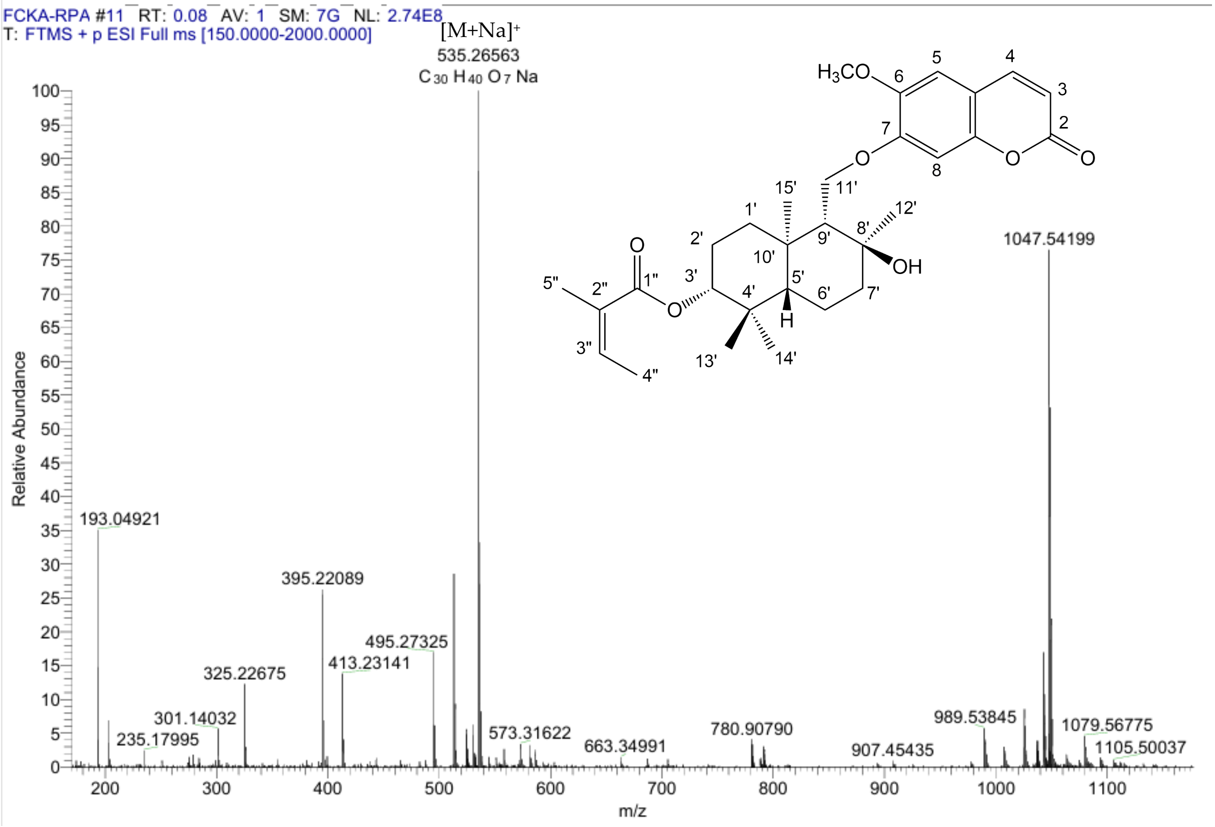

Figure S22. (+)-HRESIMS spectrum of 8′-*epi*-kayserin B angelate (**3**)

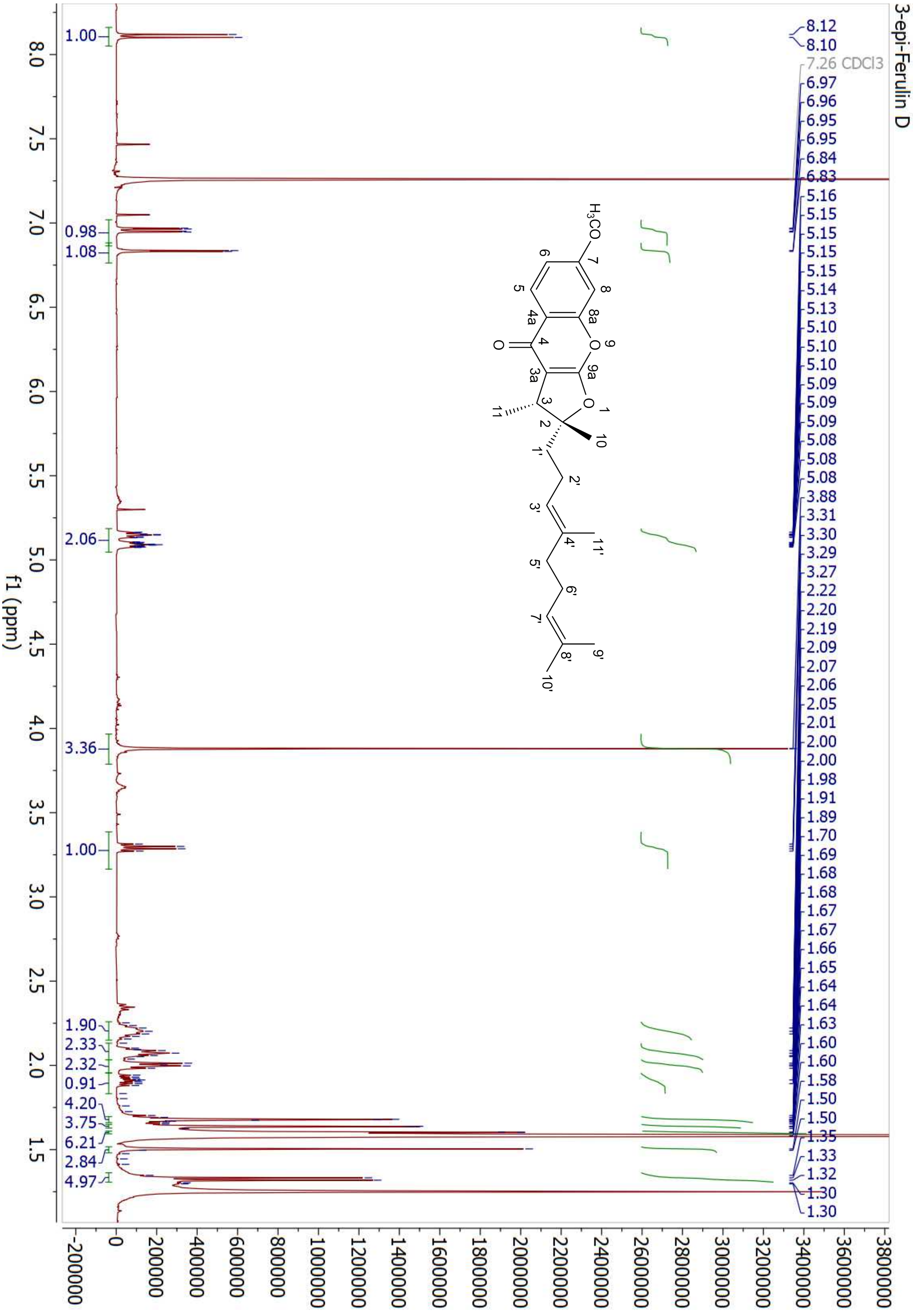

Figure S23. ^{1}H NMR spectrum (500 MHz, $CDCl_3$) of 3-*epi*-ferulin D (**4**)

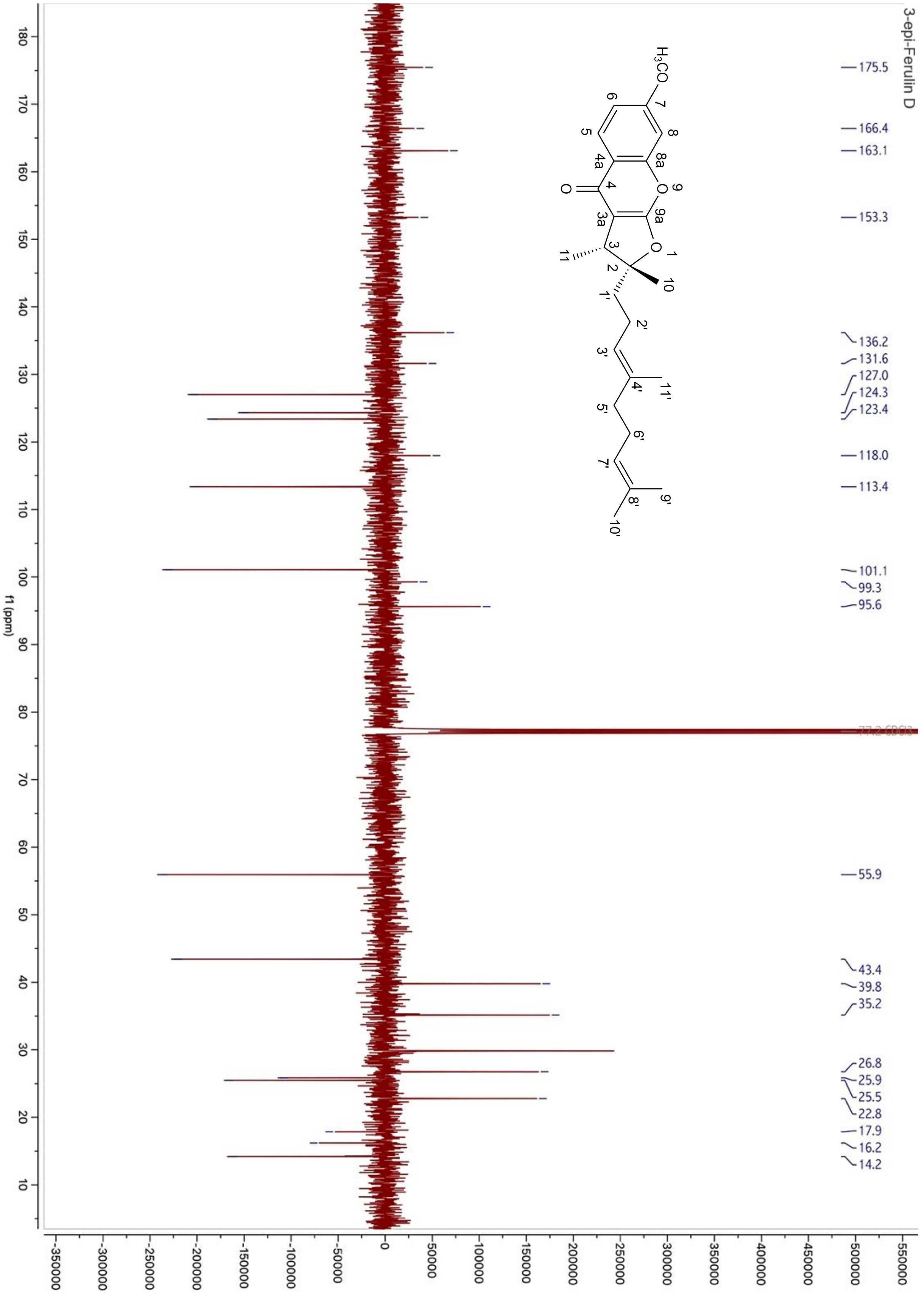

Figure S24. ^{13}C NMR spectrum (125 MHz, $CDCl_3$) of 3-*epi*-ferulin D (**4**)

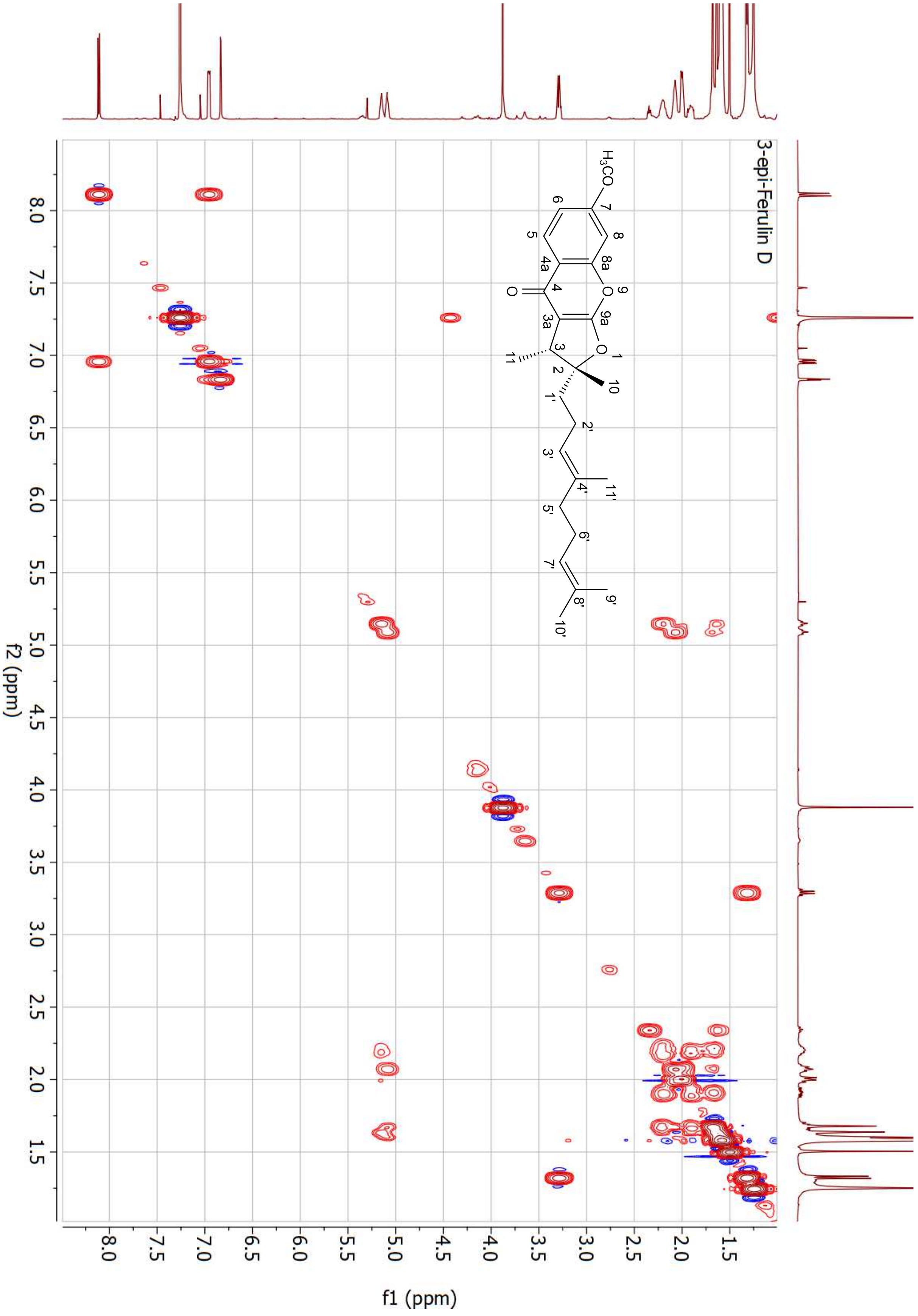

Figure S25. ^{1}H-^{1}H COSY spectrum ($CDCl_3$) of 3-*epi*-ferulin D (**4**)

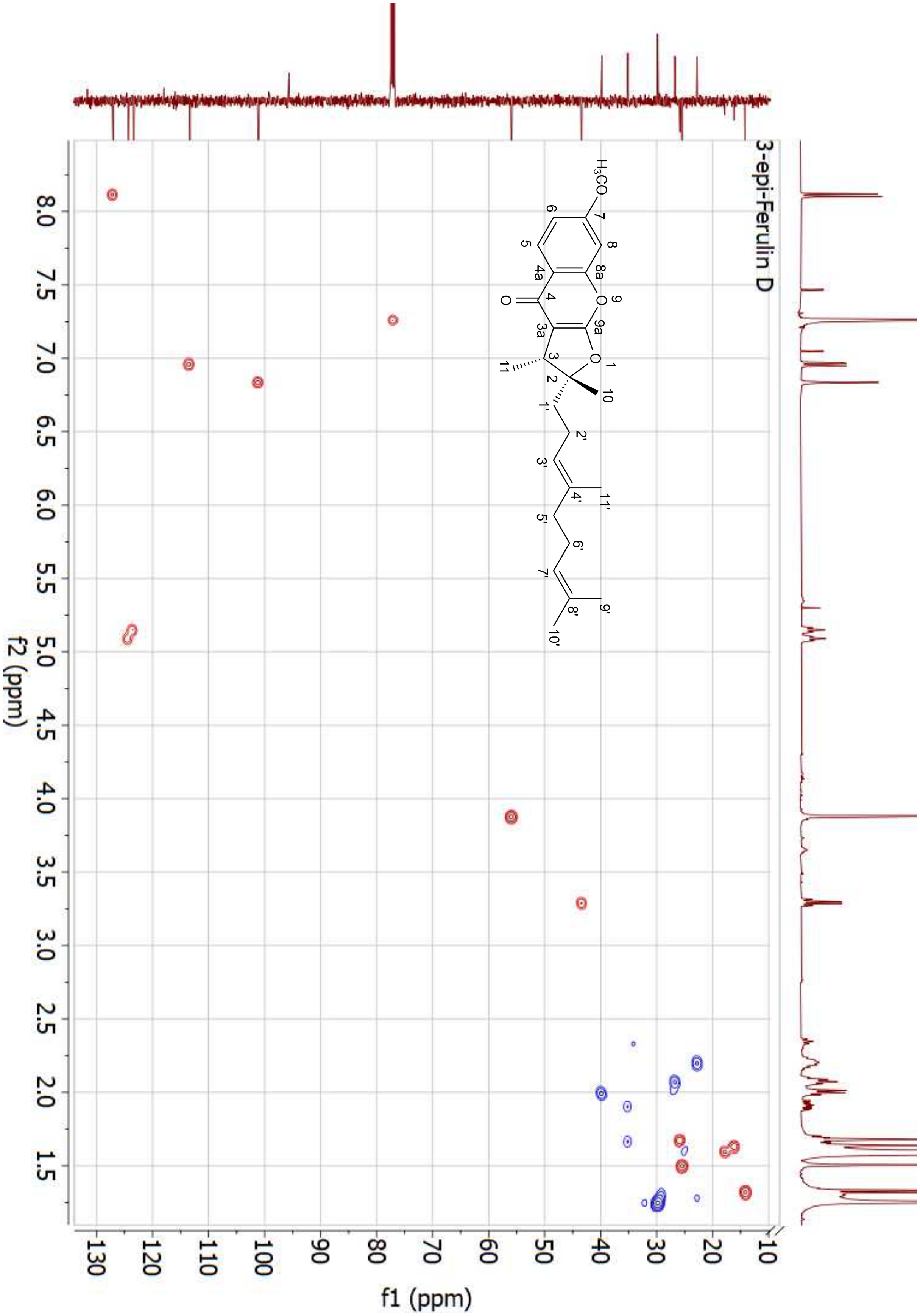

Figure S26. HSQC spectrum ($CDCl_3$) of 3-*epi*-ferulin D (**4**)

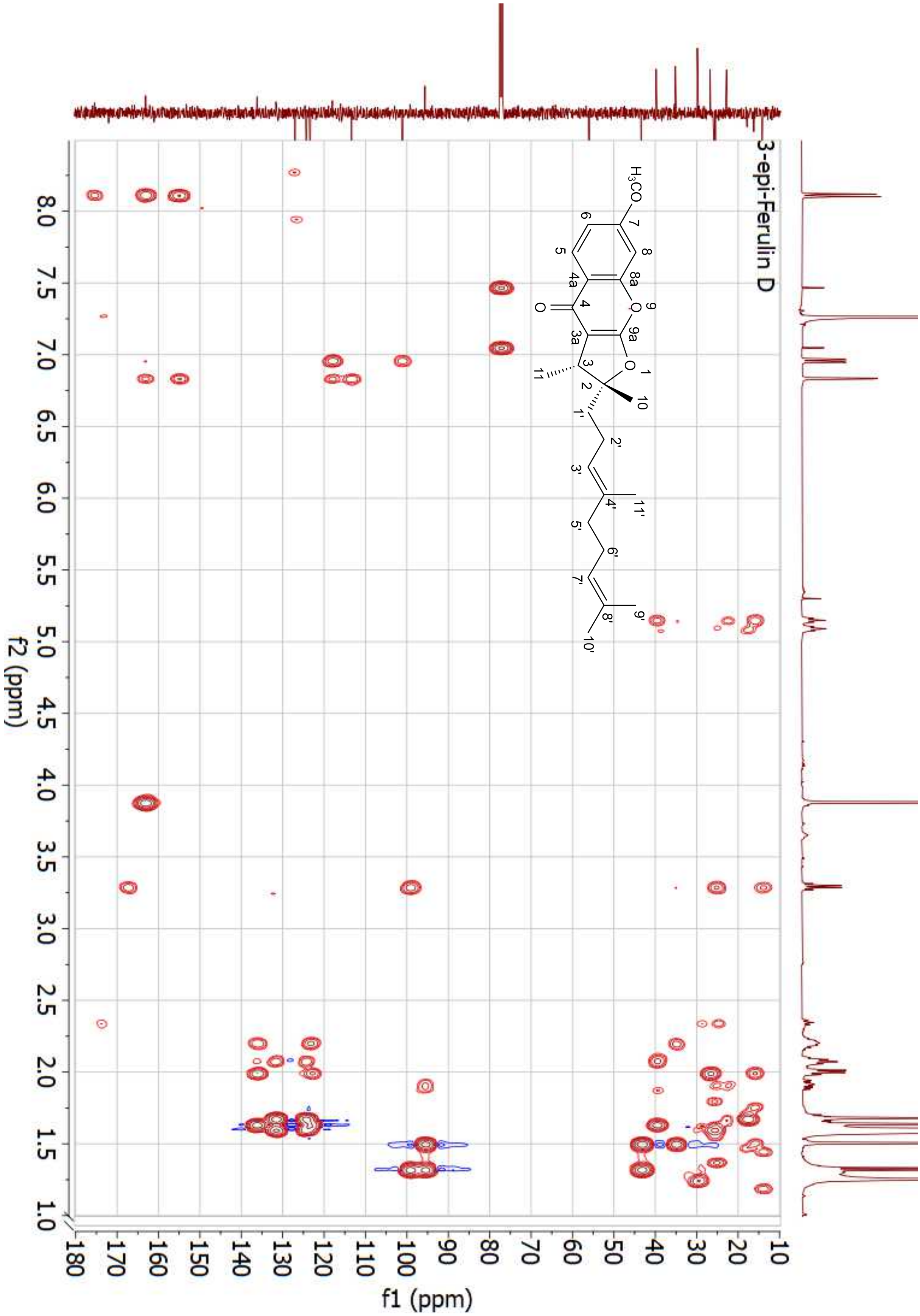

Figure S27. HMBC spectrum ($CDCl_3$) of 3-*epi*-ferulin D (**4**)

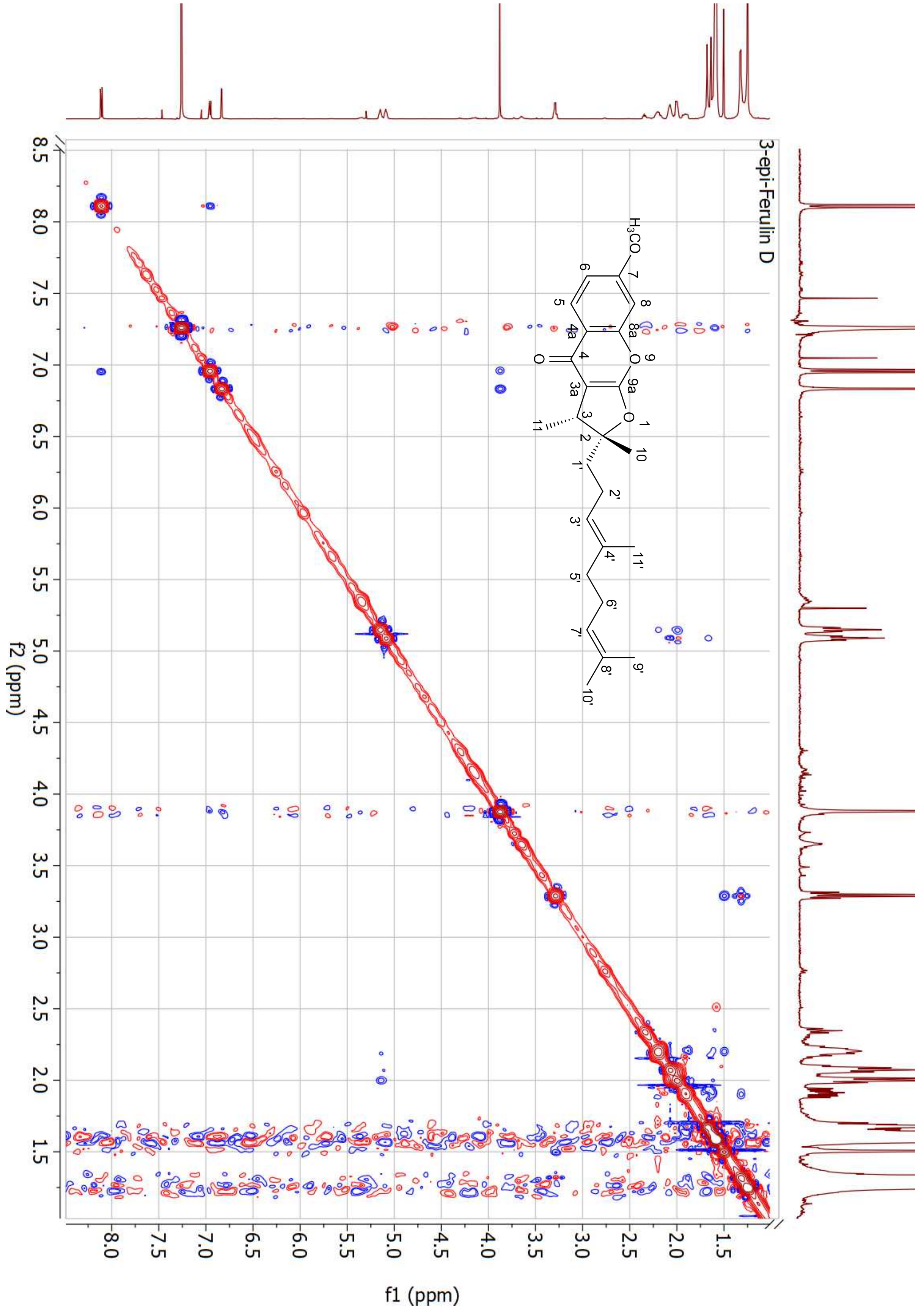

Figure S28. NOESY spectrum (CDCl3) of 3-*epi*-ferulin D (**4**)

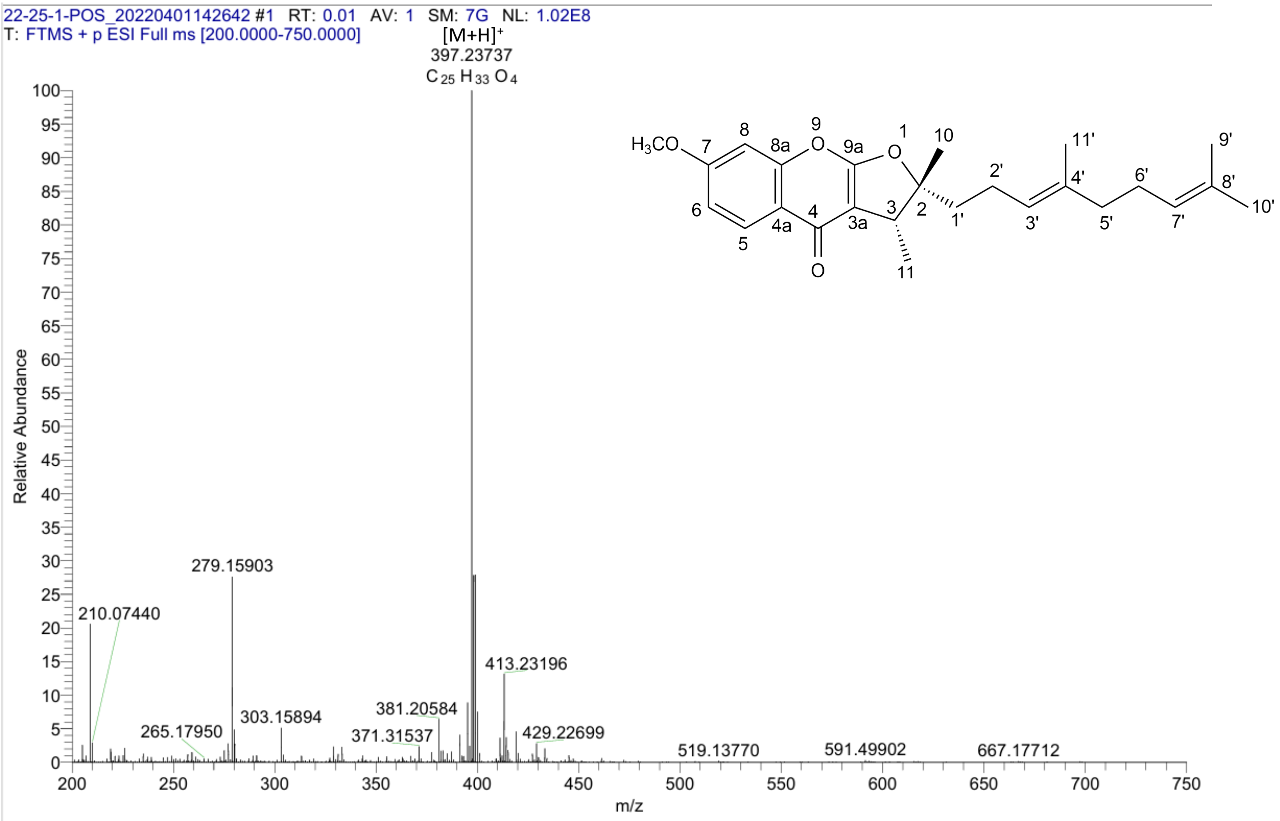

Figure S29. (+)-HRESIMS spectrum of 3-*epi*-ferulin D (**4**)

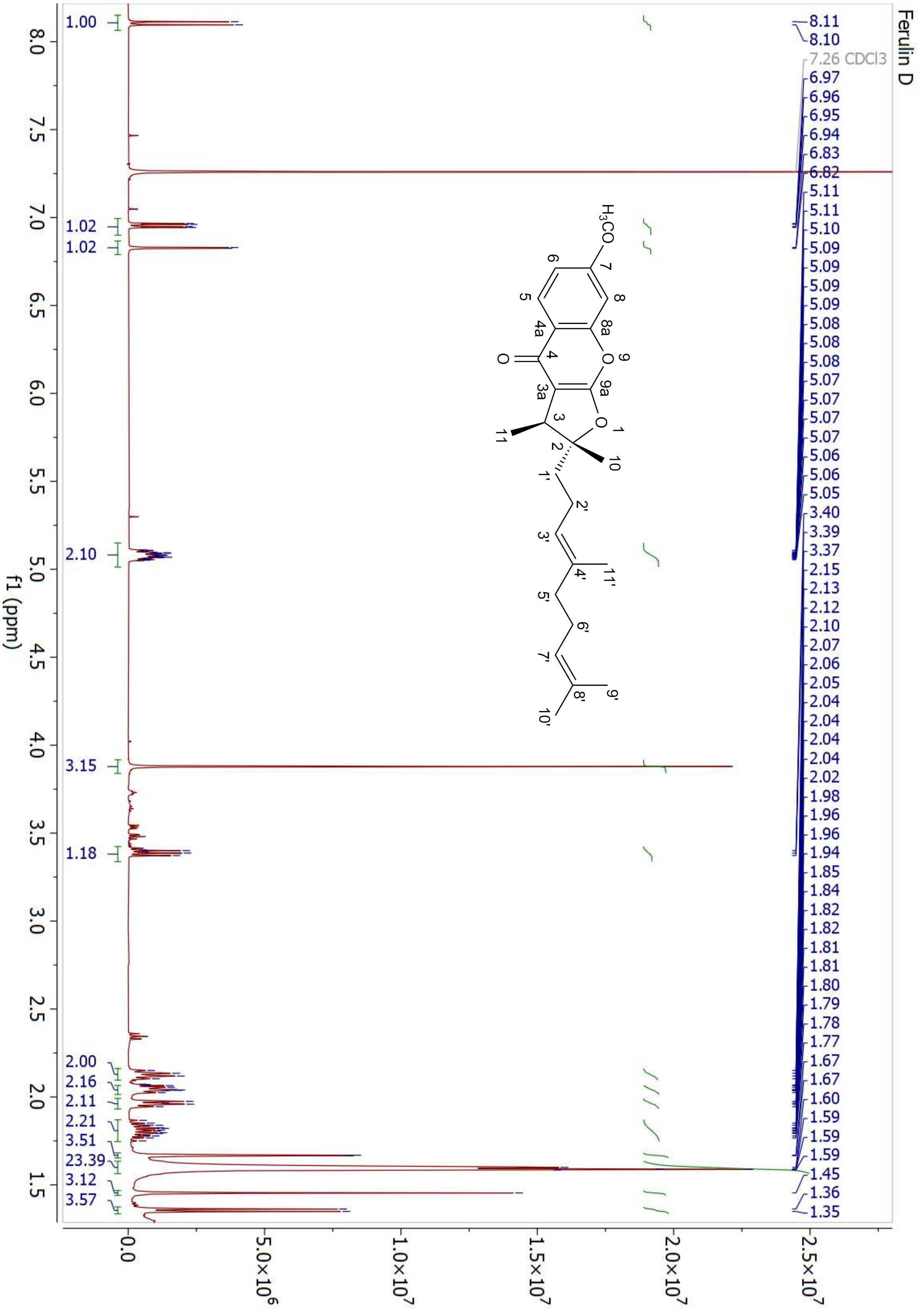

Figure S30. ^{1}H NMR spectrum (500 MHz, $CDCl_3$) of ferulin D (**5**)

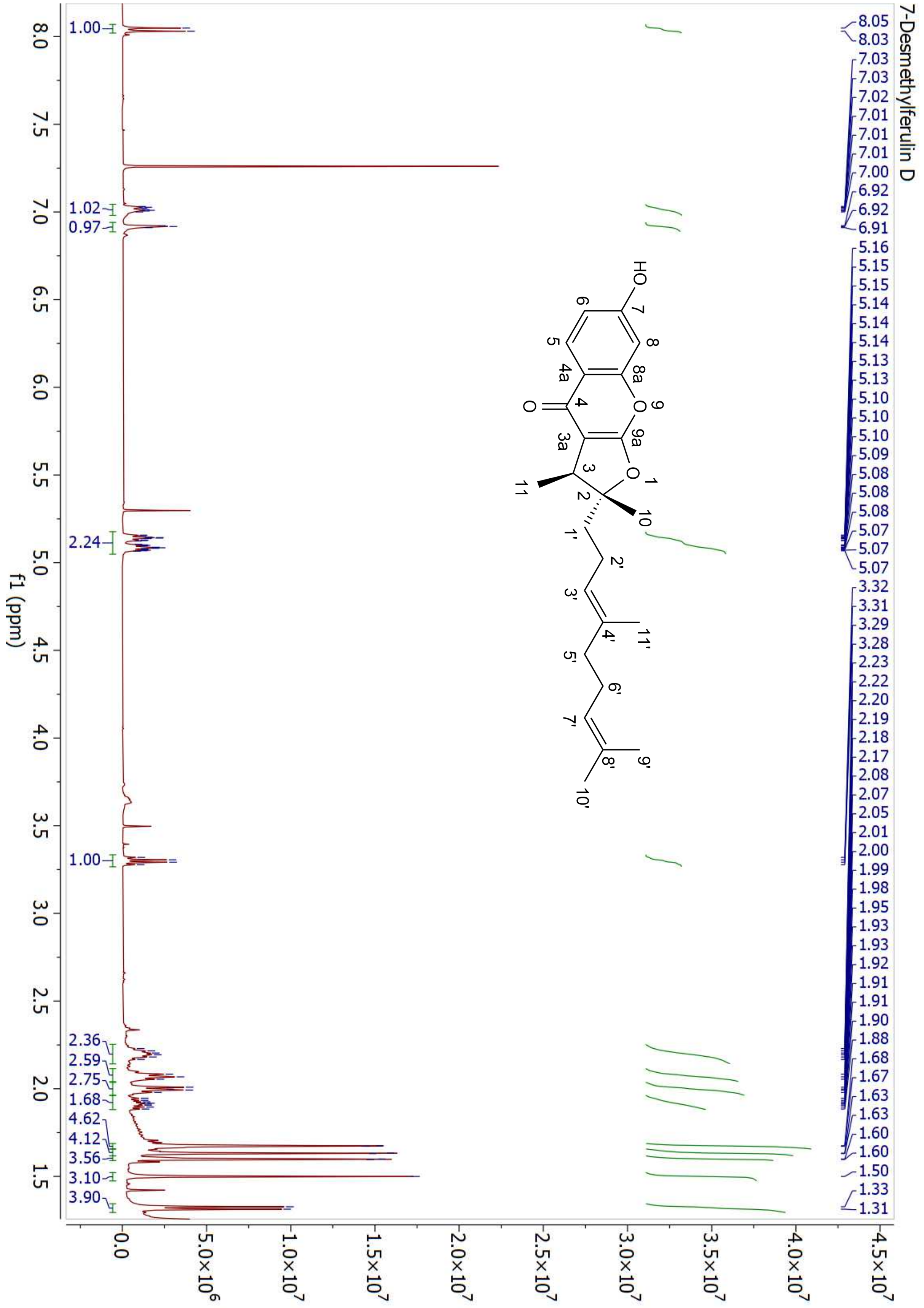

Figure S31. ^{1}H NMR spectrum (500 MHz, $CDCl_3$) of 7-desmethylferulin D (**6**)

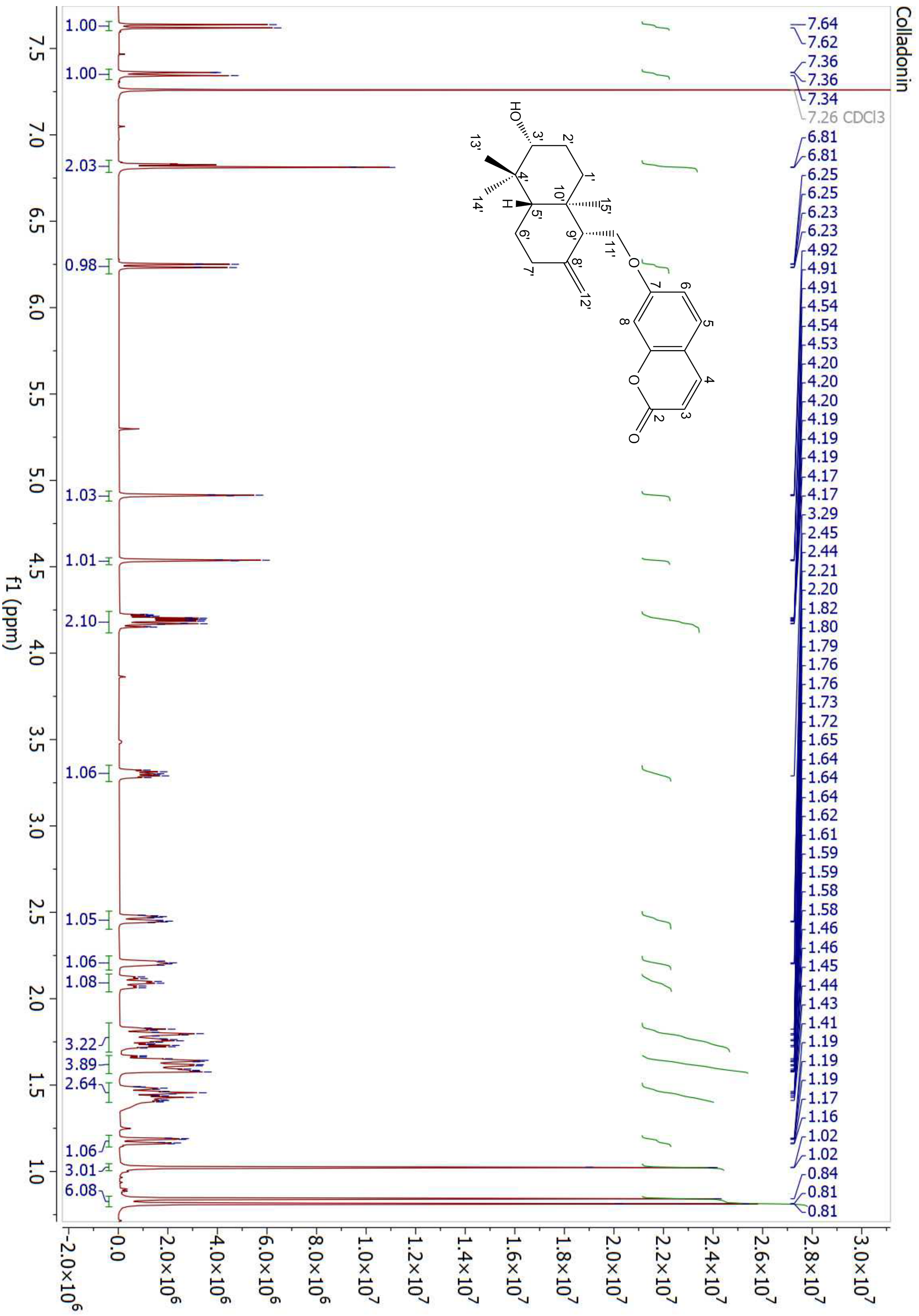

Figure S32. ^{1}H NMR spectrum (500 MHz, $CDCl_3$) of colladonin (**7**)

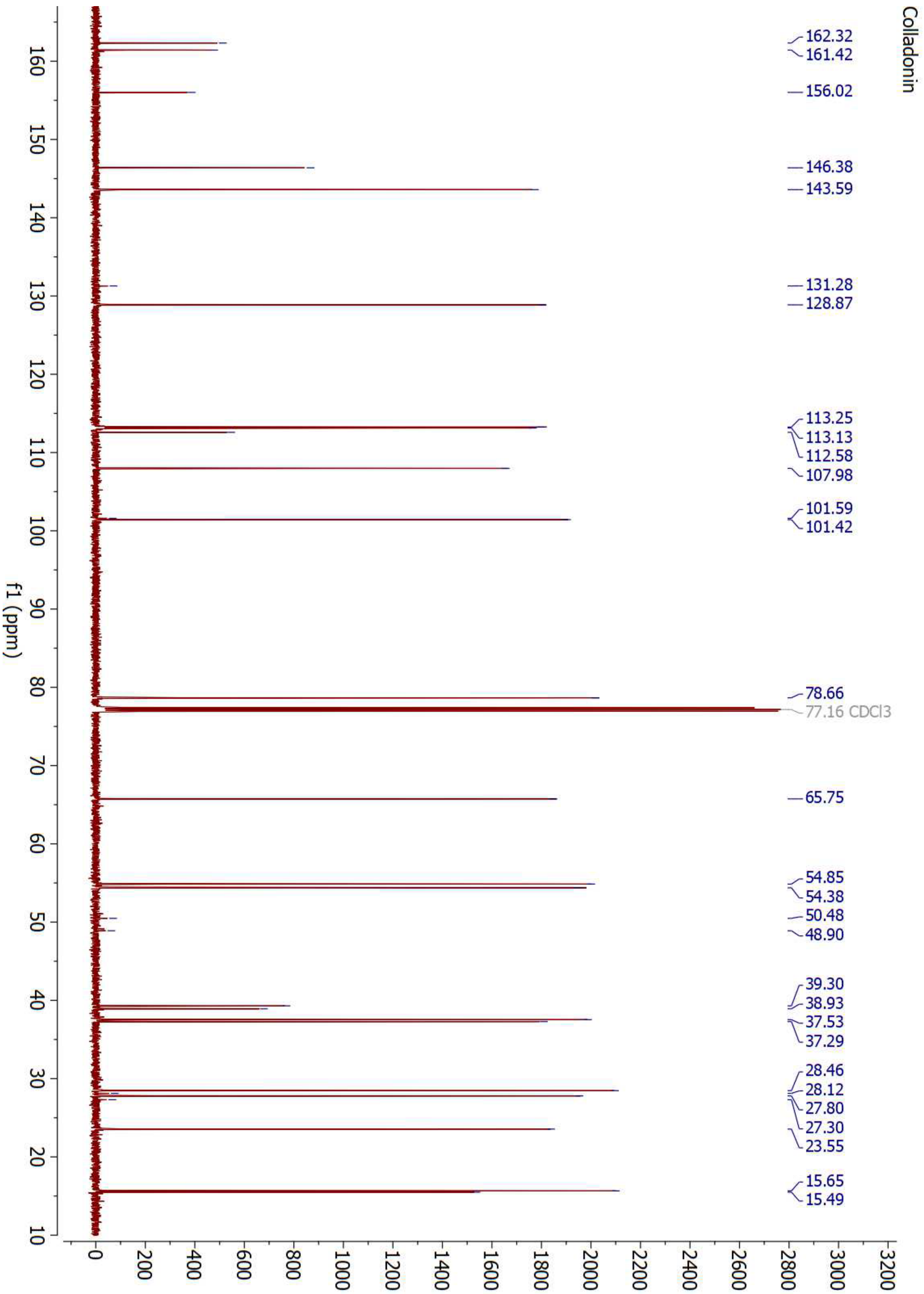

Figure S33. ^{13}C NMR spectrum (125 MHz, $CDCl_3$) of colladonin (**7**)

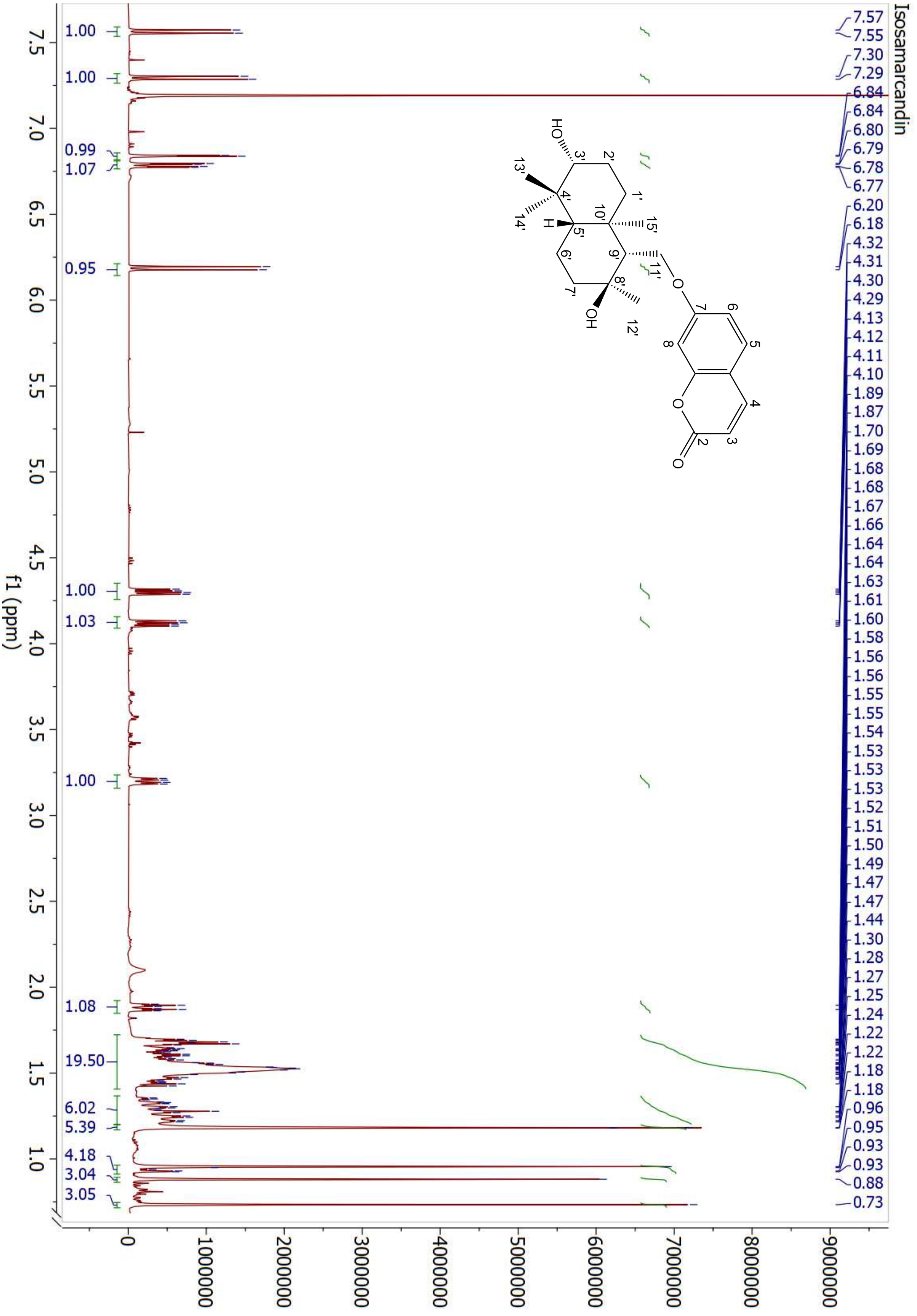

Figure S34. ^{1}H NMR spectrum (500 MHz, $CDCl_3$) of isosamarcandin (**8**)

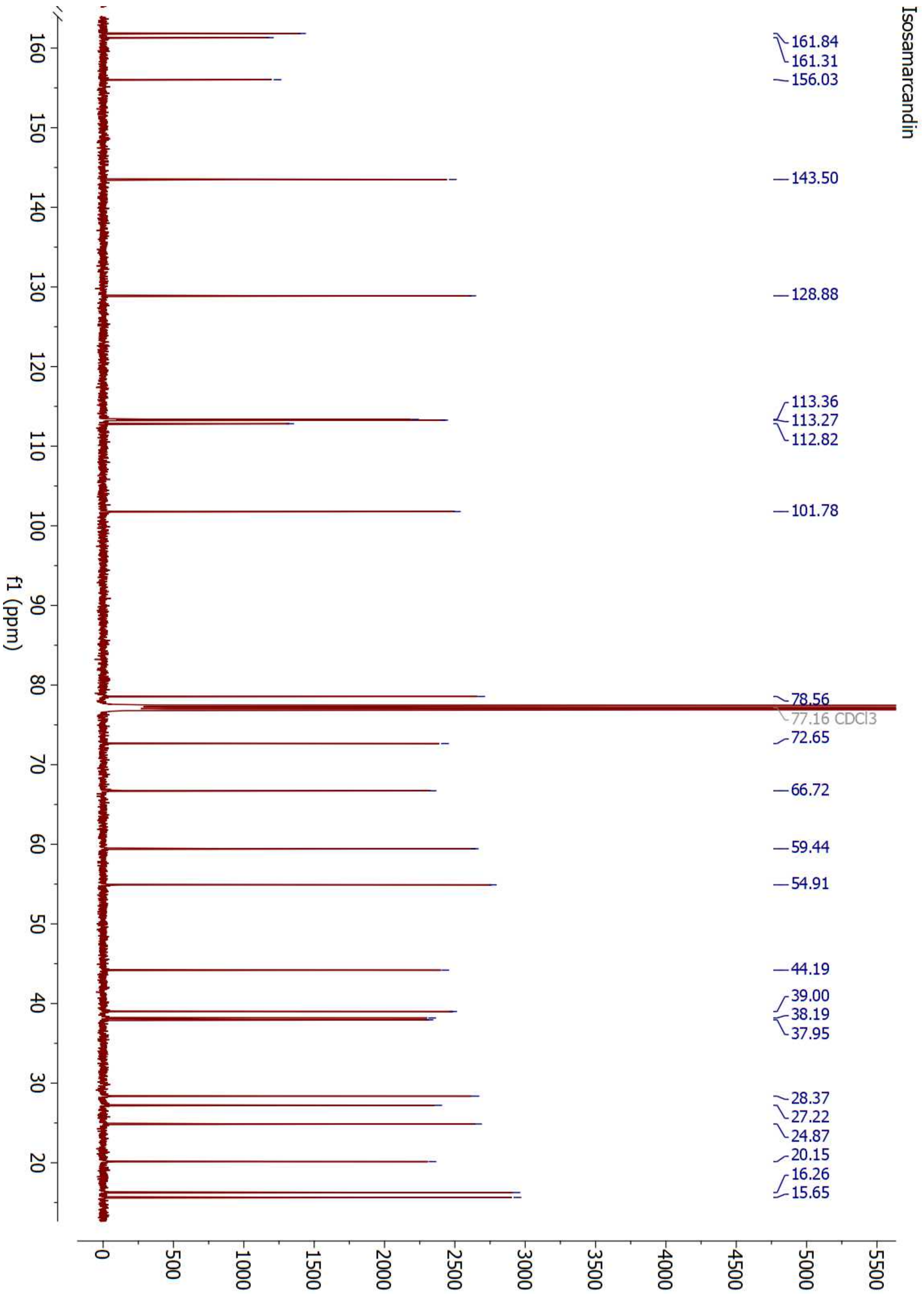

Figure S35. ${}^{13}C$ NMR spectrum (125 MHz, $CDCl_3$) of isosamarcandin (**8**)

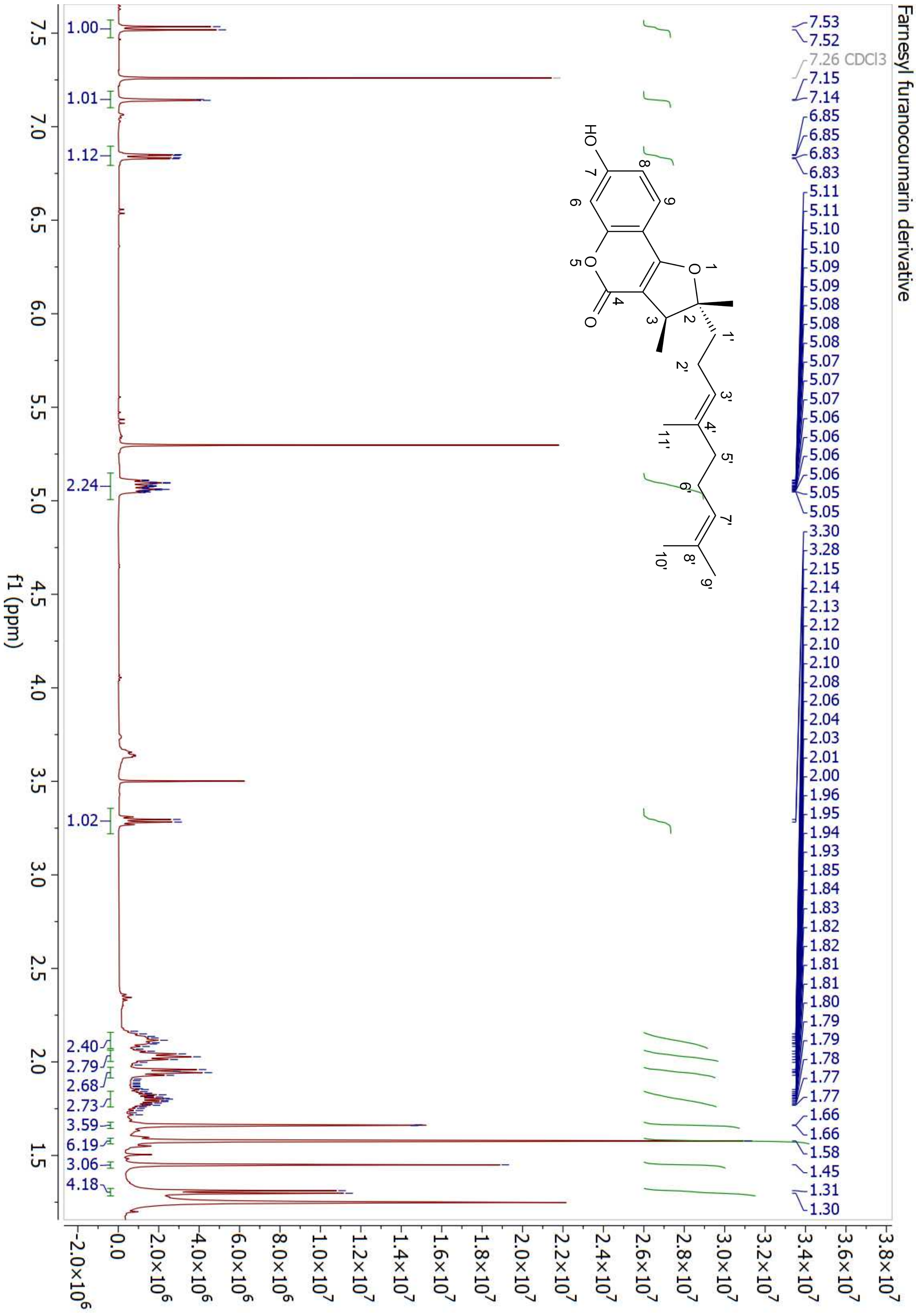

Figure S36. ^{1}H NMR spectrum (500 MHz, $CDCl_3$) of farnesyl furanocoumarin derivative (**9**)

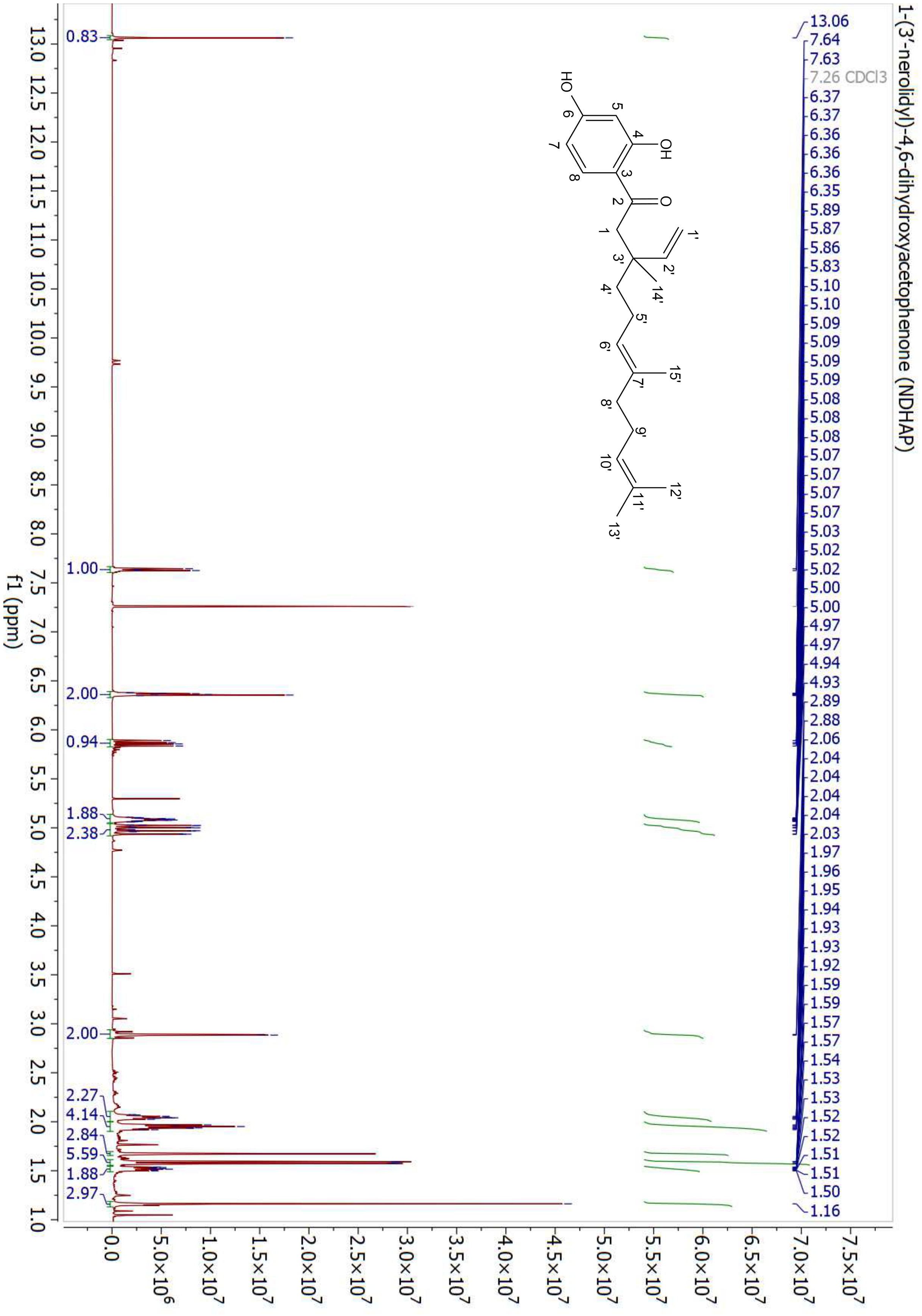

Figure S37. ^{1}H NMR spectrum (500 MHz, $CDCl_3$) of 1-(3′-nerolidyl)-4,6-dihydroxyacetophenone (NDHAP) (**10**)

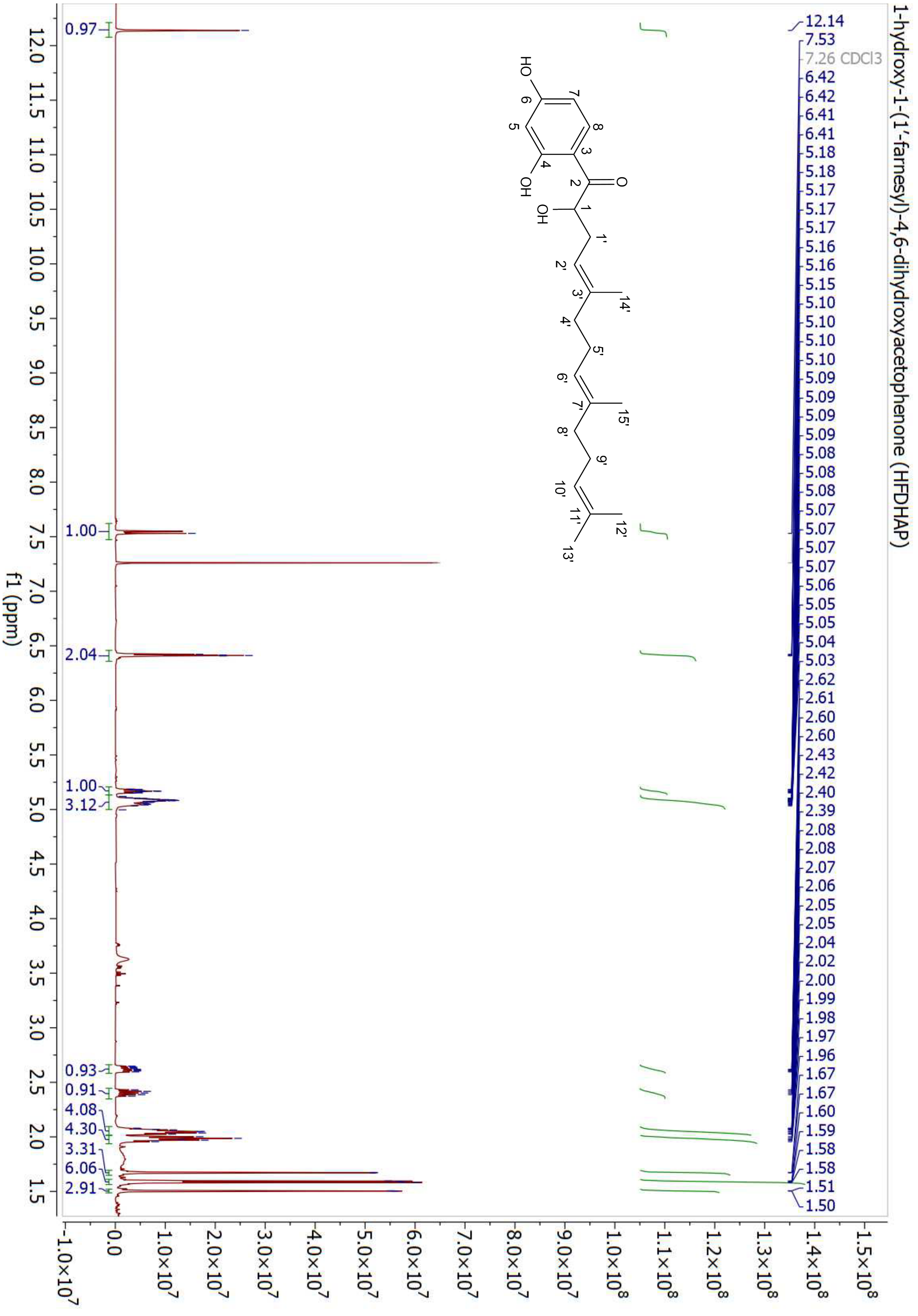

Figure S38. ^{1}H NMR spectrum (500 MHz, $CDCl_3$) of 1-hydroxy-1-(1′-farnesyl)-4,6-dihydroxyacetophenone (HFDHAP) (**11**)
